# Translational Molecular and Fluid Biomarkers for Age-Related Macular Degeneration: Practical Insights from Animal Models and Humans

**DOI:** 10.3390/biom15111571

**Published:** 2025-11-08

**Authors:** Simona Intonti, Chiara Olivieri, Michele Reibaldi, Enrico Borrelli, Claudia Curcio, Federica Maria Conedera

**Affiliations:** 1 Department of Molecular Biotechnology and Health Sciences, University of Turin, 10126 Turin, Italy; simona.intonti@unito.it (S.I.); claudia.curcio@unito.it (C.C.); 2 Molecular Biotechnology Center, University of Turin, 10126 Turin, Italy; 3 Department of Surgical Sciences, University of Turin, 10126 Turin, Italy; chiara.olivieri@unito.it (C.O.); michele.reibaldi@unito.it (M.R.); enrico.borrelli@unito.it (E.B.); 4 Department of Ophthalmology, “City of Health and Science” Hospital, 10126 Turin, Italy; 5 Department of BioMedical Research, Immunology RIA, Inselspital, University of Bern, 3008 Bern, Switzerland

**Keywords:** age-related macular degeneration, biomarkers, oxidative stress, inflammation and angiogenesis

## Abstract

Age-related macular degeneration (AMD) is a leading cause of irreversible central vision loss. Its pathogenesis is complex and multifactorial, involving genetic predisposition, inflammation, oxidative stress, and environmental influences, which underscores the need to better understand biomarkers associated with the disease. This review provides a comprehensive translational overview of biomarkers linked to both dry and wet forms of AMD by integrating findings from human studies and preclinical mouse models, including chemical, genetic, and laser-induced paradigms. It outlines key tissue, fluid, and systemic biomarkers related to oxidative stress, inflammation, complement activation, extracellular matrix remodeling, angiogenesis, and gut microbiota alterations. The main findings highlight similarities and differences between human AMD and animal models, identify challenges in biomarker validation, and emphasize the potential of combining biomarker profiles from ocular tissues, blood, tear fluid, aqueous and vitreous humor, and gut microbiome samples to improve early diagnosis, therapeutic monitoring, and personalized treatment strategies. These insights suggest that integrating experimental and clinical biomarker data could advance precision medicine in AMD, facilitating better early detection and individualized therapies. Future research should aim to bridge these datasets to optimize biomarker-driven approaches for AMD management.

## 1. Introduction

Dry age-related macular degeneration (dry AMD), the most prevalent form of AMD, represents a progressive neurodegenerative condition of the macula, affecting approximately 85–90% of AMD patients [1]. Non-exudative AMD is characterized by the gradual accumulation of drusen and progressive photoreceptor degeneration. In its advanced stage, known as geographic atrophy (GA), there is marked atrophy of the retinal pigment epithelium (RPE) and underlying choroid. GA represents a leading cause of irreversible central vision loss in the elderly population [2,3,4]. Patients with AMD may be thus classified as having early-stage disease (early/intermediate dry AMD), in which visual function is generally not affected, or late AMD (generally characterized as either “wet” neovascular AMD, “dry” atrophic AMD or both), in which central vision is severely compromised [5]. Currently, wet AMD can be managed with anti-vascular endothelial growth factor (anti-VEGF, Vascular Endothelial Growth Factor) therapies, which help control disease progression and preserve vision. Although recent FDA-approved therapies for GA, the advanced non-exudative stage of AMD, can slow disease progression, no treatments are currently available that restore vision already lost due to advanced atrophy, highlighting a critical unmet clinical need [5]. The disease arises from a complex interplay of genetic susceptibility, chronic inflammation, oxidative stress, and mitochondrial dysfunction, with strong associations identified in genome-wide studies involving variants in CFH, ARMS2/HTRA1, and C3 [6,7]. Despite the significant burden of non-exudative AMD, no approved treatments currently exist to halt or reverse disease progression in its early and intermediate stages. Although complement inhibitors have recently been approved to slow the progression of GA, the advanced form of non-exudative AMD, a critical need remains for effective therapies that restore lost vision, as well as for early diagnostic and prognostic biomarkers to improve patient outcomes. Biomarker discovery efforts in recent years have focused on a wide array of biological sources, including blood, plasma, aqueous humor, tear fluid, and gut microbiota, to identify molecular signatures associated with disease onset, progression, and therapeutic response [8,9,10]. Understanding and validating these biomarkers in both human patients and animal models will be key to advancing precision medicine and developing targeted interventions for dry AMD. The integration of genetic mouse models with the analysis of systemic biofluids and gut-derived biomarkers provides a powerful framework for studying AMD pathogenesis. These biomarkers not only validate disease mechanisms at the ocular–systemic interface but also offer translational potential for the development of early diagnostics and personalized treatment strategies.

## 2. Methods

### 2.1. Literature Search Strategy

We conducted a comprehensive literature search across PubMed, Scopus, and Web of Science databases covering the period from inception to July 2025. The search used combinations of keywords including “Age-Related Macular Degeneration,” “AMD biomarkers,” “oxidative stress,” “inflammation,” “angiogenesis,” “complement activation,” and “gut microbiota.” Both human clinical studies and preclinical animal model research (chemical, genetic, and laser-induced AMD models) were included to integrate mechanistic and translational insights.

### 2.2. Study Selection

Studies describing molecular biomarkers in ocular tissues, blood and plasma, tear fluid, aqueous and vitreous humor, as well as gut microbiome alterations associated with AMD pathogenesis and progression were selected. Original research articles, comprehensive reviews, and meta-analyses reporting validated biomarkers or novel candidate molecules relevant to early diagnosis, prognosis, or therapeutic monitoring were prioritized. Articles were excluded if they lacked relevance to AMD or did not provide sufficient methodological detail.

### 2.3. Evidence Grading

We qualitatively assessed evidence based on study design, cohort size, and biomarker validation status. Biomarkers consistently replicated across multiple independent cohorts, validated by orthogonal techniques (e.g., ELISA, proteomics, transcriptomics), or correlated with clinical severity and progression were ranked with higher evidential weight. We noted limitations in murine model fluid biomarker data and emphasized biomarkers conserved between animal models and human AMD.

### 2.4. Data Synthesis

Extracted information was synthesized in a narrative format emphasizing biomarker type, biological source, methodological approach, and relevance to AMD subtypes. We highlighted key molecular pathways including oxidative and inflammatory signaling, complement system dysregulation, extracellular matrix remodeling, angiogenic processes, and gut-retina axis contributions. Gaps in knowledge and technical challenges were identified to guide future translational research.

## 3. Preclinical Studies

### 3.1. Chemical Dry AMD Models for Mechanistic Discovery

Chemically induced AMD models are valuable for investigating disease mechanisms by selectively triggering oxidative stress, photoreceptor apoptosis, or RPE degeneration that mimic the features of dry and neovascular AMD. Sodium iodate (NaIO_3_) is widely used to induce RPE damage, secondary photoreceptor loss, and chronic inflammation, also altering miRNAs like miR-146a-5p and miR-21-5p involved in retinal inflammation and necroptosis [11]. N-methyl-N-nitrosourea (MNU) causes rapid photoreceptor apoptosis and neuroinflammation [12]. Lipofuscin components such as all-trans-retinal and A2E accumulate in the RPE, promoting oxidative stress and complement activation [13,14]. Hydrogen peroxide induces acute oxidative damage in vitro, modeling early RPE dysfunction [15]. For neovascular AMD, intraocular injections of VEGF, FGF2, or inflammatory cytokines stimulate angiogenesis and blood-retinal barrier breakdown, while cobalt chloride mimics hypoxia by stabilizing HIF-1α and inducing VEGF expression [13,16,17,18,19]. These models collectively facilitate the study of key molecular biomarkers across tissues and fluids relevant to AMD pathology. These models have several advantages, including the ability to rapidly induce disease and study the effects of oxidative stress and inflammation on retinal structure and function. However, chemically induced models primarily cause acute damage and may not accurately replicate the chronic, multifactorial pathophysiology of human dry AMD, particularly with regard to the disease’s progressive and age-related nature. Furthermore, pathological features such as drusen formation, geographic atrophy progression and systemic metabolic alterations are not fully mirrored by these models. Despite these limitations, chemically induced models remain valuable tools for screening candidate therapeutics, investigating acute molecular pathways and establishing biomarkers in preclinical research [20].

#### 3.1.1. Retina and RPE—Choroid Tissue Markers in Chemical Models

**Figure 1:**
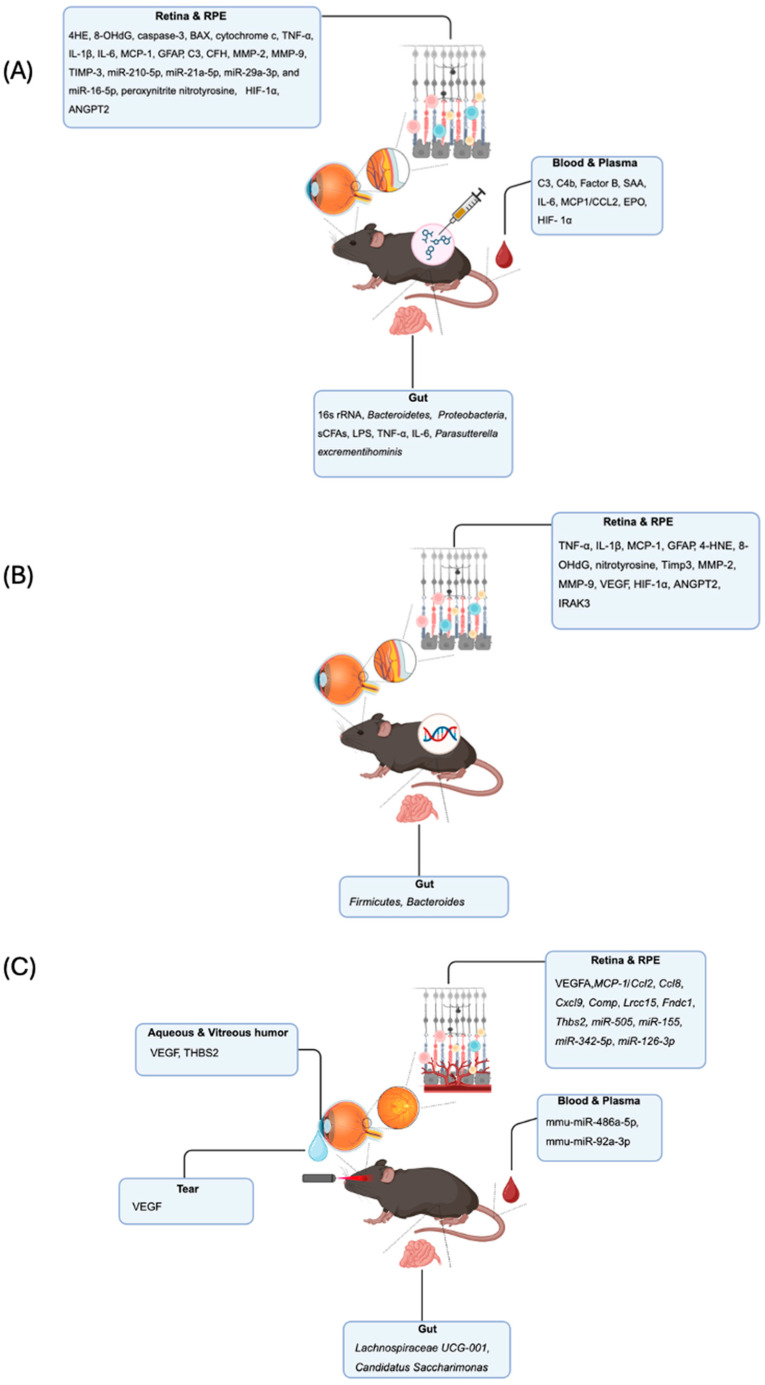
**Overview of AMD preclinical models and associated biomarkers.** (**A**) Chemically induced dry AMD mouse models, (**B**) genetically engineered dry AMD mouse models, and (**C**) laser-induced wet AMD mouse models.

In chemically induced models of AMD, including those using NaIO_3_, MNU, atRAL, and bis-retinoids such as A2E, extensive analyses of retina and RPE–choroid complexes have revealed multiple biomarkers indicative of oxidative stress, inflammation, cell death, and extracellular matrix remodeling. In NaIO_3_-treated rodents, oxidative stress markers such as 4-hydroxynonenal (4-HNE), 8-hydroxy-2′-deoxyguanosine (8-OHdG), and nitrotyrosine are consistently upregulated in RPE and photoreceptor layers, indicating ROS-mediated cellular injury [21] (Figure 1A). Apoptotic markers, including cleaved caspase-3, Bcl-2-associated X protein and cytochrome c, are also elevated, particularly in the outer nuclear layer and RPE, reflecting photoreceptor degeneration [12,22]. Inflammatory mediators such as TNF-α, IL-1β, IL-6, Monocyte Chemoattractant Protein (MCP)-1, and Glial Fibrillary Acidic Protein (GFAP) (indicative of Müller cell gliosis) are increased in both NaIO_3_ and MNU models [23] (Figure 1A). In models using A2E or atRAL, accumulation of these bis-retinoids in RPE cells leads to lysosomal dysfunction and upregulation of complement components like C3 and CFH, as well as lipid peroxidation products, closely mimicking the chronic features of dry AMD [24]. Additionally, altered expression of structural and extracellular matrix proteins such as MMP-2, MMP-9, and TIMP-3 has been observed, reflecting Bruch’s membrane (BrM) remodeling and RPE–choroid barrier disruption [21,23] (Figure 1A). Additional models employing CoCl_2_, a chemical hypoxia mimetic, simulate ischemic damage and HIF-1α pathway activation, leading to VEGF upregulation, glial activation, and neuronal loss in the outer retina [25] (Figure 1A). Notably, CoCl_2_ exposure alters the expression of several microRNAs (miRNAs) involved in the regulation of oxidative stress, angiogenesis, and cell survival. Specifically, miR-210-5p, miR-21a-5p, miR-29a-3p, and miR-16-5p are significantly upregulated, while miR-183-5p is downregulated, highlighting the contribution of non-coding RNA networks to hypoxia-induced retinal damage [26] (Figure 1A). Notably, miR-21 is predominantly upregulated in RPE cells, where modulates necroptosis; miR-183 is chiefly expressed in retinal neurons, whereas miR-210 is induced in photoreceptors and retinal vascular endothelial cells under hypoxic conditions [27,28,29]. Similarly, exogenous administration of 4-HNE or H_2_O_2_ directly induces oxidative stress in retinal explants or in vivo, resulting in mitochondrial dysfunction, RPE barrier disruption, and increased expression of pro-apoptotic proteins and pro-inflammatory [30]. Additionally, these stressors modulate autophagic flux and have been used to study the interplay between oxidative damage and defective clearance mechanisms in AMD. Altered expression of structural and ECM proteins such as MMP-2, MMP-9, and Tissue Inhibitor of Metalloproteinase (TIMP)-3 has been consistently observed across models, reflecting BrM remodeling and RPE–choroid barrier disruption [30,31]. In the VEGF-induced model, localized overexpression of VEGF leads to RPE and photoreceptor layer disorganization, increased levels of the peroxynitrite nitrotyrosine, infiltration of CD68^+^ macrophages, upregulation of HIF-1α, ANGPT2, and MMP-9 in the choroid and retina [32,33,34] (Figure 1A).

#### 3.1.2. Measurable Systemic Biomarkers in Chemical Models

In the NaIO_3_ model, which induces selective RPE damage and secondary photoreceptor loss, systemic analyses of plasma have identified a distinct inflammatory signature. Proteomic and cytokine profiling revealed increased levels of complement proteins (C3, C4b, factor B) and serum amyloid A (SAA), indicative of innate immune activation [31]. Concurrently, elevated plasma concentrations of IL-6 and MCP-1/CCL2 reflect a chronic inflammatory state associated with retinal degeneration [35] (Figure 1A). Similarly, in CoCl_2_-treated animals plasma levels of VEGF, IL-6, and erythropoietin (EPO) are significantly upregulated, suggesting systemic responses to oxidative stress and Hypoxia-Inducible Factor (HIF)-1α pathway activation [36] (Figure 1A).

#### 3.1.3. Directly Measurable Microbiome Changes in Chemical Models

Emerging evidence from chemically induced models of AMD, particularly those using NaIO_3_ or oxidative stress-inducing agents, highlights the gut–retina axis as a contributor to retinal degeneration. In NaIO_3_-treated mice, 16S rRNA sequencing of fecal samples revealed pronounced gut dysbiosis, characterized by a decrease in *Firmicutes* and an increase in *Bacteroidetes* and *Proteobacteria*, microbial shifts commonly linked to pro-inflammatory states [37]. This imbalance was associated with reduced levels of short-chain fatty acids (SCFAs), especially butyrate, and elevated concentrations of lipopolysaccharide (LPS) in both stool and plasma, suggesting increased intestinal permeability and systemic inflammation. These changes correlated with retinal thinning and enhanced expression of inflammatory cytokines such as IL-6 and TNF-α in the retina [37] (Figure 1A). Therapeutic interventions using probiotics or SCFA supplementation have demonstrated retinal protective effects, supporting a functional link between gut microbial metabolites and retinal integrity [38]. Notably, in the NaIO_3_-induced mouse model, combined treatment with *Lactobacillus fermentum* NS9 and *Aronia anthocyanidin extract* (AAE) produced beneficial effects both at the retinal level and in modulating gut microbiota composition. Metagenomic analysis showed a significant enrichment of the genus *Parasutterella*, particularly *P. excrementihominis*, in the AAE+LF group compared to both the untreated and AAE-only groups (Figure 1A). *Parasutterella* has been previously associated with immunomodulatory activity and the metabolism of bile acids and amino acids, and its increased abundance may contribute to reduced oxidative stress and systemic inflammation [37].

### 3.2. Genetic Mouse Models for Dry AMD: Pathogenic Mechanisms and Biomarker Potential

Understanding the complex pathophysiology of AMD has greatly advanced through the development of genetically engineered mouse models that mimic key features of both dry and neovascular AMD. These models enable detailed investigation of the molecular and cellular processes underlying disease onset and progression, particularly within the retina and RPE-choroid complex. Inflammation-driven models such as CC chemokine ligand 2 (Ccl2)^−^/^−^, C-X3-C Motif Chemokine Receptor 1 (Cx3cr1)^−^/^−^, and Ccl2^−^/^−^Cx3cr1^−^/^−^ replicate aspects of dry AMD by exhibiting subretinal microglial accumulation, upregulation of pro-inflammatory cytokines, and complement activation [39,40,41]. Oxidative stress models, including Sod1^−^/^−^ mice, demonstrate how impaired antioxidant defense leads to RPE degeneration and photoreceptor loss through increased production of oxidative biomarkers such as 4-HNE and 8-OHdG [42,43,44]. Meanwhile, models involving HtrA1 overexpression and Timp3 mutations (e.g., S179C) highlight the role of extracellular matrix remodeling in AMD pathogenesis [45,46,47]. Importantly, emerging evidence from C3-deficient and Complement factor H (CFH)-variant mouse models suggests a functional link between AMD-associated genetic variants and alterations in the gut microbiota, reinforcing the role of systemic factors in modulating retinal disease [48,49]. Collectively, these models provide a valuable experimental framework for dissecting the multifactorial nature of AMD and for identifying novel therapeutic targets across inflammatory, oxidative, angiogenic, and systemic pathways. Despite their usefulness, genetically engineered mouse models have both strengths and limitations. The main advantage of these models is the ability to manipulate specific genes in order to elucidate causal mechanisms and to recreate distinct pathological features of dry AMD in a controlled environment. These models are also employed in the preclinical testing of potential drugs and to study cell–cell interactions within the retinal microenvironment. However, significant differences in ocular anatomy and lifespan between mice and humans limit the direct application of findings, particularly with regard to the macula, which is absent in rodents. Furthermore, most models only reproduce selected aspects of AMD, rather than the complete disease spectrum that evolves over decades in humans. Nevertheless, these models remain fundamental resources for unraveling disease mechanisms, identifying therapeutic targets, and advancing translational research in the AMD field [50].

#### 3.2.1. Retina and RPE-Choroid Alteration in Genetic Models

Genetically engineered mouse models of AMD have provided valuable insights into molecular and cellular alterations occurring within the retina and RPE–choroid complex during disease progression. In Ccl2^−^/^−^, Cx3cr1^−^/^−^, and Ccl2^−^/^−^ Cx3cr1^−^/^−^ mice, which exhibit features reminiscent of dry AMD, histological and transcriptomic analyses revealed elevated expression of inflammatory markers such as TNF-α, IL-1β, and MCP-1 in the RPE and subretinal space, along with microglial accumulation and complement activation [40,41,51] (Figure 1B). Increased GFAP expression indicates Müller cell gliosis, while upregulation of Iba1 and CD68 highlights enhanced microglial activity and infiltration. In Sod1^−^/^−^ mice, characterized by impaired oxidative defense, the retina shows elevated levels of oxidative stress biomarkers including 4-HNE, 8-OHdG, and nitrotyrosine, particularly in the photoreceptor layer and RPE, correlating with progressive degeneration [43,44] (Figure 1B). Liu et al. [52] identified interleukin-1 receptor–associated kinase M (IRAK-M) as a critical immunoregulatory protein in the RPE that progressively declines with age. Rare genetic variants in IRAK3, the gene encoding IRAK-M, were found to be associated with an increased risk of developing AMD (Figure 1B). Analyses of human tissues and genetic mouse models revealed that IRAK-M expression in the RPE diminishes with aging and oxidative stress, with a more pronounced reduction in AMD-affected tissues. Mice lacking Irak3 exhibited early-onset outer retinal degeneration, which was further aggravated by oxidative insults. The loss of IRAK-M disrupted RPE homeostasis, leading to mitochondrial dysfunction, cellular senescence, and abnormal cytokine production [52]. Conversely, IRAK-M overexpression conferred protection to RPE cells against oxidative and immune stress [52]. Notably, subretinal delivery of adeno-associated virus (AAV) expressing human IRAK3 mitigated light-induced retinal damage in wild-type mice and reduced age-related degeneration in Irak3-deficient mice. These findings suggest that restoring IRAK-M levels in the RPE may help counteract chronic inflammation and cellular dysfunction in AMD, offering a promising therapeutic strategy for retinal degeneration. The studies involving IRAK-M utilized a rather unique light toxicity model. The rationale for this model is that light exposure induces acute oxidative and immune stress in the retinal pigment epithelium, simulating environmental risk factors for AMD and allowing the assessment of IRAK-M’s role in regulating retinal homeostasis and vulnerability. However, the model represents an acute injury rather than the chronic and gradual progression typical of human AMD. Moreover, the intensity of light exposure does not reflect normal physiological aging conditions. These limitations should be considered when interpreting the results [53]. Models overexpressing HtrA1 or carrying disease-associated mutations in Timp3 (e.g., S179C) display thickening of BrM, accumulation of basal deposits, and upregulation of extracellular matrix remodeling enzymes such as MMP-2 and MMP-9 in BrM [46,54] (Figure 1B).

Despite the extensive use of genetically engineered mouse models to study the molecular and cellular mechanisms of AMD, there is a notable lack of published data on body fluid biomarkers, particularly in the context of these models. Specifically, blood and plasma biomarkers, such as complement components, inflammatory cytokines, or oxidative stress markers, are rarely measured or reported in a systematic manner. Rodents exhibit genetic, structural, and functional differences in key complement proteins and their regulators, which affect the localization, expression, and activation pathways of complement components. These interspecies disparities reduce the translational comparability of rodent data to human AMD and likely contribute to the limited focus on complement biomarkers in murine studies. Such differences must be carefully considered when interpreting complement-related data and when developing complement-targeted therapies based on mouse models. Similarly, tear fluid biomarkers remain virtually unexplored in murine models due to technical limitations in sample collection and the small volume of tears in rodents. Additionally, aqueous and vitreous humor biomarkers, which are clinically relevant in human AMD for tracking intraocular inflammation, angiogenesis, and complement activity, are seldom analyzed in mouse models, largely because of the challenges associated with fluid extraction from small eyes. This gap in biomarker profiling in murine systems limits the ability to directly translate findings from genetic models to clinical diagnostics and hinders the development of non-invasive monitoring strategies in preclinical AMD research. Addressing this deficiency through improved micro-sampling techniques and proteomic or transcriptomic analyses could substantially enhance the translational value of these models.

#### 3.2.2. Measurable Systemic and Microbiome Biomarkers from Genetic Models

Zysset-Burri et al. [55] demonstrated that complement C3-deficient mice exhibited a higher Firmicutes-to-Bacteroides ratio compared to wild-type controls, a microbial profile resembling that commonly observed in patients with nAMD (Figure 1B). In human studies, the CFH3 single nucleotide polymorphism (SNP) was significantly associated with nAMD and positively correlated with increased abundance of *Negativicutes*. Additionally, individuals carrying the CFH3 variant showed elevated levels of *Clostridiales*, whereas those with CFH1 and CFH2 variants exhibited a negative correlation with this microbial group. These findings support a functional interaction between host genetics and the gut microbiome in AMD, suggesting that genetically driven alterations in microbial composition may influence complement system activation and contribute to disease progression [48,56]. A deeper understanding of this gene–microbiota interplay will be essential for developing personalized therapeutic strategies targeting AMD pathogenesis.

### 3.3. Laser-Induced Neovascularization Wet AMD Model: Translational Biomarker Discovery

The laser-induced neovascularization model is the most widely used experimental approach for studying wet AMD [57,58]. In this model, laser burns are applied to the mouse retina using a focused green Argon laser or a similar photocoagulation system to rupture BrM, which triggers the growth of abnormal new blood vessels from the choroid into the subretinal space [59]. As a result, subretinal neovascularization develops, often accompanied by vascular leakage and local inflammation [60]. This process closely mimics key pathological hallmarks of human wet AMD, such as MNV, vascular leakage, and subretinal fibrosis, making the model highly relevant for investigating disease mechanisms and testing new therapies [61,62].

The main advantages of the laser-induced neovascularization model are its reproducibility, rapid induction of lesions, and compatibility with a wide range of genetic backgrounds and imaging techniques [63]. However, it also has limitations: it represents an acute injury rather than the slow, age-related progression seen in human AMD, and mice lack a macula, the central retinal region most affected in patients [60,64]. Despite these differences, the model reliably recapitulates the angiogenic, inflammatory, and fibrotic responses central to human wet AMD, and has been instrumental in the preclinical development of anti-VEGF therapies and the identification of disease biomarkers, supporting its continued relevance for translational AMD research [65].

Because the laser-induced neovascularization closely mirrors the molecular and cellular changes seen in human AMD, it serves as a powerful platform for biomarker discovery. Using this model, researchers have identified a range of tissue and fluid biomarkers that reflect disease activity and offer insights into underlying mechanisms. The sections below highlight key biomarkers identified in the retina, blood, tear fluid, ocular humors, and even the gut microbiome, emphasizing their relevance for translational AMD research.

#### 3.3.1. Retina and RPE Biomarkers in Laser-Induced Models

Laser-induced neovascularization in mice leads to profound molecular changes in the retina and RPE-choroid complex. Transcriptomic and proteomic analyses reveal upregulation of angiogenic genes such as VEGFA, a primary driver of neovascularization and a direct target of anti-VEGF therapies in humans [66]. Inflammatory genes, including Ccl2 (MCP-1), Ccl8, and Cxcl9, are also highly expressed, reflecting immune cell recruitment and a pro-inflammatory microenvironment [67,68] (Figure 1C). These responses are not unique to mice; comparative studies show that many of these genes are conserved and similarly upregulated in human AMD lesions, underscoring their translational relevance [61]. Additionally, extracellular matrix remodeling genes such as Comp, Lrcc15, Fndc1, and Thbs2 are elevated, contributing to fibrosis, a process that can limit the effectiveness of anti-angiogenic treatments and lead to vision loss in patients [68]. MiRNAs including miR-505, miR-155, miR-342-5p, and miR-126-3p are also differentially expressed, acting as post-transcriptional regulators of these pathogenic pathways [32] (Figure 1C). The identification of these conserved molecular signatures supports the model’s use for identifying new therapeutic targets and for understanding the mechanisms underlying neovascularization and fibrosis in AMD.

#### 3.3.2. Directly Measurable Serum and Plasma Biomarkers

In the laser-induced neovascularization mouse model, the search for systemic, minimally invasive biomarkers has focused on circulating miRNAs due to their stability and regulatory roles in angiogenesis and inflammation. Recent studies have identified robust dysregulation of mmu-miR-486a-5p and mmu-miR-92a-3p in both the blood and RPE/choroid tissues following laser-induced neovascularization, as confirmed by RNA sequencing and RT-qPCR [69] (Figure 1C). These miRNAs are functionally relevant, with evidence that mmu-miR-486a-5p and mmu-miR-92a-3p modulate microglial cell viability and mobility, processes implicated in neovascularization pathogenesis [69]. Importantly, similar miRNA alterations have been reported in plasma samples from patients with neovascular AMD, highlighting their translational potential as biomarkers for disease activity, early detection, and therapeutic monitoring [69]. The accessibility of blood sampling further supports the clinical utility of these biomarkers, allowing for longitudinal assessment without invasive ocular procedures. Additionally, panels of miRNAs may improve diagnostic accuracy beyond individual markers. The conservation of miRNA dysregulation between the laser-induced neovascularization and human AMD underscores the value of this model for biomarker discovery and validation in translational research [69].

#### 3.3.3. Biomarkers Directly Measurable in Tear Fluid

Tear fluid has recently emerged as a valuable, non-invasive source of biomarkers for neovascular AMD. Studies using the mouse model have demonstrated that VEGF concentrations are significantly increased in tears following neovascularization induction, mirroring findings in human patients with active neovascular AMD [70] (Figure 1C). The primary source of this tear VEGF is the choroid-RPE complex, and its presence in tears reflects ongoing angiogenic and inflammatory activity within the eye. The ease and repeatability of tear sampling make it particularly attractive for longitudinal disease monitoring and assessing therapeutic response. Notably, clinical studies have also reported that tear VEGF levels can differ by sex, suggesting potential for personalized biomarker strategies. However, current findings remain at the discovery stage, being primarily hypothesis-generating based on results from small preclinical and clinical cohorts. Mouse model studies show increased tear VEGF after neovascularization induction, paralleling findings from exploratory human cohorts. Despite this, reproducibility constraints stemming from limited sample size and methodological differences necessitate caution in interpretation. To advance clinical utility, further validation is required, including standardized tear collection, diurnal control, and multicenter patient cohorts using targeted quantification (e.g., PRM, ELISA) with pre-registered endpoints. The parallel elevation of tear VEGF in both the murine model and human AMD underscores the translational promise of tear-based assays, which could facilitate routine, non-invasive monitoring of disease activity and treatment efficacy in clinical practice [70].

#### 3.3.4. Directly Measurable Aqueous and Vitreous Humor Biomarkers

The analysis of aqueous and vitreous humor has provided important insights into intraocular biomarkers for neovascular AMD. In the laser-induced neovascularization model, levels of angiogenic and fibrotic proteins such as VEGF and thrombospondin 2 (THBS2) are significantly elevated following neovascularization induction [71] (Figure 1C). These proteins are well-established mediators of pathological neovascularization and tissue remodeling in AMD. Measurement of VEGF and related factors in ocular fluids is already integrated into clinical practice, particularly for patients undergoing intravitreal injections or ocular surgery. The ability of the murine model to recapitulate these biomarker changes further validates its utility for preclinical evaluation of anti-angiogenic and anti-fibrotic therapies. Moreover, the parallel findings in both animal models and human patients support the ongoing development of aqueous and vitreous fluid-based biomarkers for monitoring disease activity and therapeutic response in neovascular AMD [61,72].

#### 3.3.5. Directly Measurable Microbiome Biomarkers

During neovascularization, marked shifts in gut bacterial composition—particularly increases in *Lachnospiraceae* UCG-001 and *Candidatus Saccharimonas*—have been observed, correlating with distinct alterations in fecal metabolite profiles [73] (Figure 1C). Of note, members of the *Lachnospiraceae* family, known producers of SCFAs, can regulate host immune responses and preserve epithelial barrier integrity [74] (Figure 1C).

The gut–retina axis thus emerges as a promising avenue for biomarker discovery and therapeutic development. Although the clinical translation of stool-based biomarkers in AMD remains at an early stage, current evidence underscores the systemic nature of ocular diseases and highlights the potential of gut microbiome profiling for risk assessment, disease monitoring, and microbiome-targeted intervention [75].

Collectively, these findings indicate that the laser-induced neovascularization model not only reproduces key aspects of human neovascular AMD but also serves as a robust platform for identifying and validating clinically relevant biomarkers across multiple tissues and biofluids, advancing mechanistic insight and translational research in AMD [69,70].

## 4. Human Studies

### 4.1. Biomarkers in “Dry” AMD in Humans: From Tissue to Biofluids

Dry AMD, also known as non-neovascular or atrophic AMD, is the most prevalent form of the disease, accounting for up to 90% of cases. It primarily affects individuals over the age of 60 and is characterized by a gradual loss of central vision due to progressive degeneration of the macula, the central region of the retina responsible for detailed visual tasks [5]. A hallmark of early dry AMD is the accumulation of extracellular deposits known as drusen, which form between the RPE and BrM. These deposits, composed of lipids, proteins, and complement factors, are thought to arise from impaired fluid and metabolite transport across BrM, leading to metabolic stress in the RPE. The pathogenesis of dry AMD begins with a thickening of BrM, primarily due to the buildup of lipid- and protein-rich material. This accumulation disrupts the normal exchange of nutrients and waste products between the choroid and the retina, compromising RPE homeostasis. As a result, oxidative stress increases within RPE cells, leading to the accumulation of lipofuscin, an autofluorescent byproduct of incomplete lysosomal degradation, that further impairs lysosomal function and cholesterol metabolism. In response to cellular stress, RPE cells may release membranous vesicles such as exosomes, which are believed to contribute to sub-RPE deposit formation. Moreover, the presence of serum-derived proteins in drusen suggests that systemic factors can infiltrate the sub-RPE space, likely due to BrM dysfunction, further promoting deposit accumulation [5]. Over time, these pathogenic processes can lead to GA, the advanced form of dry AMD, which involves the irreversible loss of RPE cells, photoreceptors, and the underlying choriocapillaris. This results in permanent central vision loss, for which no approved curative treatments currently exist [76]. Given the absence of approved treatments for dry AMD, particularly in advanced stages, there is a growing emphasis on the identification of early and predictive biomarkers that can aid in diagnosis, monitor disease progression, and guide therapeutic development.

#### 4.1.1. Non-Directly Measurable Tissue Mechanistic: Markers from Pathology

**Figure 2:**
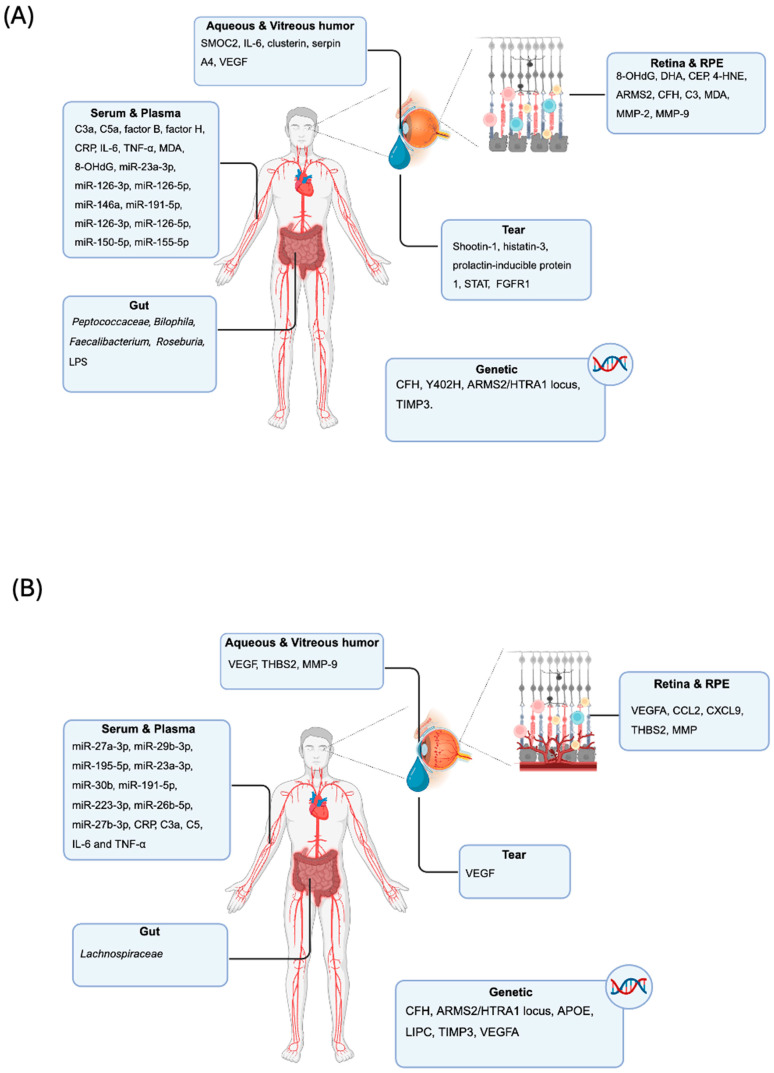
**Overview of AMD human studies and associated biomarkers.** (**A**) Dry AMD; (**B**) wet AMD.

Extensive molecular profiling of the retina and RPE-choroid complex in human dry AMD has identified numerous tissue-level biomarkers reflecting key pathogenic processes, such as oxidative stress, inflammation, lipid metabolism and extracellular matrix remodeling. Clinical studies involving AMD patients and post-mortem donor eyes have provided strong evidence for the accumulation of oxidative stress-related DNA, protein, and lipid biomarkers in AMD pathology. Among these, oxidative DNA damage, particularly the formation of 8-OHdG, has been prominently observed in AMD donor eyes, with higher levels detected in cases of dry AMD with RPE atrophy [77] (Figure 2A). Given the abundance of docosahexaenoic acid (DHA) in the lipid-rich outer segments of photoreceptors [78], oxidative degradation of DHA has been implicated in AMD, as demonstrated by increased levels of carboxyethyl pyrrole (CEP) protein adducts, a marker of DHA peroxidation, within BrM of AMD donor eyes [79,80] (Figure 2A). Notably, CEP levels were shown to be approximately 60% higher in dry AMD eyes compared to controls, as measured by ELISA [81,82]. However, the levels of 4-hydroxy-2-nonenal (4-HNE) modified proteins did not differ significantly across retinal regions or among the histological stages of AMD [83]. A separate study integrating proteomic and genomic analyses found that increased plasma CEP levels in AMD were associated with elevated expression of associated with age-related maculopathy susceptibility 2 (ARMS2), CFH, and complement C3, further linking oxidative stress to genetic susceptibility in dry AMD [84] (Figure 2A). In addition to ocular findings, systemic markers of oxidative stress such as malondialdehyde (MDA), 8-OHdG, and protein carbonyls were significantly elevated in the serum of AMD patients, suggesting that oxidative damage in AMD is not confined to the retina but reflects a broader systemic oxidative imbalance [85] (Figure 2A). Additionally, impaired extracellular matrix (ECM) turnover and thickening of BrM are early hallmarks of AMD, closely associated with altered activity of MMPs. Analyses of human AMD eyes have demonstrated elevated expression and accumulation of MMP-2 and MMP-9 in BrM and RPE choroid tissue, accompanied by deposit buildup beneath the RPE, evidence of disrupted ECM remodeling [6,86] (Figure 2A). MMP-2, primarily secreted by RPE cells, becomes dysregulated in AMD, leading to excessive collagen IV accumulation and basal deposits [86]. Although total levels of activated MMP-2 and MMP-9 may decrease in AMD donor eyes, an increase in high-molecular-weight proenzyme complexes likely hinders their activation, contributing to ECM thickening [87]. Collectively, these findings highlight the critical disruption of MMP-mediated ECM turnover as a key tissue biomarker and potential therapeutic target. Oxidative stress markers detected at the tissue level, including modifications such as 8-OHdG and CEP protein adducts in the retina and RPE, have been widely reported in AMD. These changes reflect localized oxidative damage contributing to cellular dysfunction. However, the interpretation of these tissue oxidative markers is limited by technical variability, sample heterogeneity, and difficulties in standardizing collection and processing protocols. Therefore, further studies with improved reproducibility and spatial resolution will be critical to clarify the specific pathological roles of these modifications in AMD progression [88,89].

#### 4.1.2. Directly Measurable Serum and Plasma Biomarkers

In AMD, increasing evidence suggests that systemic biomarkers detectable in blood and plasma reflect underlying pathogenic processes such as complement activation, oxidative stress, and chronic inflammation. Among the most consistently reported are components of the complement system, including elevated levels of C3a, C5a, and factor B, as well as reduced levels or functional impairment of CFH, particularly in individuals carrying the AMD-associated CFH Y402H polymorphism [90,91]. Inflammatory markers such as C-reactive protein (CRP), IL-6, and TNF-α are also elevated in the plasma of patients with dry AMD and have been linked to disease progression and genetic [92] (Figure 2A). Oxidative stress-related plasma biomarkers, including malondialdehyde (MDA), 8-OHdG, and CEP-adducts, are found at significantly higher levels in dry AMD patients compared to age-matched controls, indicating systemic lipid peroxidation and DNA damage [79,93] (Figure 2A). Furthermore, additional serum markers such as various cytokines, miRNAs, and other inflammatory proteins have been increasingly recognized for their diagnostic and prognostic relevance. For instance, circulating miRNAs including miR-23a-3p, miR-126-3p, miR-126-5p, miR-146a, miR-191-5p, as well as decreased levels of miR-16-5p, miR-17-3p, and miR-17-5p have been identified as differentially expressed in peripheral blood nuclear cells from AMD patients and controls, providing insight into systemic regulatory pathways involved in AMD pathogenesis. Multivariate analysis revealed that dry AMD was an independent factor associated with increased expression of miR-23a-3p, miR-126-3p, miR-126-5p, miR-146a, miR-191-5p and decreased expression of miR-16-5p, miR-17-3p, miR-17-5p. Six miRNAs were differentially expressed in peripheral blood nuclear cells of AMD subtypes: four were increased in dry AMD patients (miR-126-3p, miR-126-5p, miR-150-5p, miR-155-5p) whereas two were increased in wet AMD patients (miR-30b, miR-191-5p) [94]. Visual acuity and expression of miR-126-3p, miR-126-5p, miR-155-5p were positively correlated, whereas visual acuity and miR-191-5p expression were negatively correlated [94,95,96] (Figure 2A). In addition, miR-27a-3p was suggested as a potential diagnostic biomarker for both wet and dry AMD in whole blood [94].

These circulating biomarkers not only provide insights into systemic disease mechanisms but may also serve as accessible, non-invasive tools for risk stratification, early detection, and monitoring of therapeutic response in dry AMD. Systemic oxidative stress markers such as malondialdehyde (MDA), 8-OHdG, and CEP-adducts have been extensively studied in circulating fluids of AMD patients. While older studies commonly showed elevated levels, recent evidence synthesis underscores that replication is mixed across well-controlled modern cohorts, largely due to variable storage conditions, batch effects, and clinical confounders. Importantly, after comprehensive multivariable adjustment, some markers demonstrate robust associations whereas others show inconsistent results, highlighting the need for rigorous multicenter validation and standardized protocols. Thus, while promising, these circulating biomarkers require cautious interpretation and further validation before they can be reliably used for diagnosis or prognosis [97,98].

#### 4.1.3. Directly Measurable Tear Fluid Biomarkers

Tear fluid biomarkers have emerged as a promising, non-invasive source for detecting molecular changes associated with AMD. A study from Winiarczyk et al. [99] analyzed tear films from a total of 22 patients (8 with wet AMD, 6 with dry AMD, and 8 control individuals). 2D electrophoresis was used to separate tear film proteins prior to their identification with matrix-assisted laser desorption/ionization time of flight spectrometer (MALDI-TOF/TOF). They observed that dry AMD patients showed 97 specific proteins related to 44 pathway patterns and that these proteins were involved in more pathways than those identified in the wet AMD [99]. The two most widely expressed pathways were the Wnt signaling pathway and the Huntington disease pathway. It is noteworthy that there was a major representation of proteins involved in oxidative stress, inflammation, and proteolysis, e.g., the autophagy-related PI3K pathway. Autophagy failure has been reported to be associated with AMD development [100]. Notably, eight proteins, including shootin-1, histatin-3, and prolactin-inducible protein 1, were uniquely upregulated in AMD tears, suggesting their potential as disease-specific biomarkers [99] (Figure 2A). These proteins are involved in pathways related to inflammation, apoptosis, and angiogenesis, reflecting underlying AMD pathophysiology. The study also highlighted the presence of signaling molecules such as STAT3 and FGFR1 linked to neovascular processes in wet AMD [99,101] (Figure 2A). Mass spectrometry-based proteomics has further advanced tear biomarker discovery, enabling sensitive and quantitative profiling of tear proteins in ocular diseases including AMD [102]. However, current evidence remains confined to discovery-stage proteomics. The referenced study by Winiarczyk et al., although insightful, involved a limited cohort and relied on 2D-GE/MALDI-TOF, a technique suitable for exploratory screening but constrained by sample size and reproducibility. The identification of proteins linked to oxidative stress, inflammation, and autophagy provides valuable hypotheses, yet these findings require further verification and clinical validation. Advancing toward clinical applicability will demand targeted quantitation (e.g., PRM/MRM or ELISA), standardized tear collection protocols, and adequately powered multicenter studies with pre-registered statistical endpoints [102]. These findings underscore the utility of tear fluid proteomics for early diagnosis, monitoring disease progression, and evaluating therapeutic response in AMD.

#### 4.1.4. Directly Measurable Aqueous and Vitreous Humor Biomarkers

Aqueous and vitreous humor biomarkers have gained increasing attention in dry AMD research due to their proximity to retinal tissues and potential to reflect local pathological changes. Proteomic analyses of aqueous humor from AMD patients have identified significant alterations in proteins involved in inflammation, extracellular matrix remodeling, and immune regulation. For example, Huang et al. [103] used affinity-based proteomics to discover 82 proteins significantly altered in geographic atrophy (GA), a late dry AMD form, highlighting Secreted Modular Calcium-binding protein 2 (SMOC2) and IL-6 as promising biomarkers linked to RPE dysfunction and inflammation (Figure 2A). Other studies confirmed elevated levels of complement and coagulation cascade proteins, such as clusterin and serpin A4, in aqueous humor, in dry AMD patients suggesting dysregulated protein metabolism and immune activation [104] (Figure 2A). Additionally, increased VEGF levels have been detected in aqueous humor of dry AMD patients with subretinal drusen deposits, implicating angiogenic signaling even in early disease [105]. Although vitreous humor is less frequently studied in dry AMD, proteomic profiling indicates changes in complement factors and neurodegeneration-related proteins, supporting its role as a biomarker source [106]. Collectively, aqueous and vitreous humor biomarker studies provide valuable insights into local molecular alterations in dry AMD, offering potential for improved diagnosis and monitoring of disease progression.

#### 4.1.5. Directly Measurable Microbiome Biomarkers

Emerging evidence from recent studies supports a significant association between gut microbiota composition and dry AMD. Metagenomic and mendelian randomization analyses have identified specific microbial taxa causally associated with disease susceptibility, with *Peptococcaceae*, *Bilophila*, *Faecalibacterium*, and *Roseburia* linked to increased risk, while *Candidatus Soleaferrea*, *Desulfovibrio*, and the *Eubacterium ventriosum* group appear protective [107] (Figure 2A). Mechanistically, dysbiosis may promote increased gut permeability and elevated LPS translocation, triggering chronic low-grade inflammation that affects retinal health. In parallel, metabolomic profiling of stool samples from dry AMD patients reveals altered levels of microbial metabolites—including bile acids as already reported in chemical mouse models, tryptophan catabolites, and branched-chain amino acids—implicated in immune modulation and oxidative stress pathways relevant to AMD pathogenesis [55,107,108].

### 4.2. Biomarkers in “Wet” AMD in Humans: Clinical and Biofluids Applications

Neovascular, or wet AMD is a leading cause of irreversible central vision loss in the elderly population worldwide. The disease is characterized by the growth of abnormal choroidal blood vessels through BrM into the subretinal space, resulting in exudation, hemorrhage, and ultimately fibrotic scarring that damages the macula [109]. While anti-VEGF therapies have transformed the management of wet AMD, inter-individual variability in disease progression and treatment response remains a significant challenge. This has driven the search for robust biomarkers that can support early diagnosis, predict prognosis, guide therapeutic decisions, and monitor disease activity. In humans, biomarker discovery has leveraged advances in ocular imaging, molecular profiling of ocular and systemic fluids, and genetic analysis, providing a multidimensional understanding of disease pathogenesis and progression.

#### 4.2.1. Retinal Imaging Markers and Tissue Pathology

Direct sampling of retinal and RPE-choroid tissues in human patients is limited to rare surgical specimens or postmortem studies, but advances in non-invasive imaging have enabled the identification of surrogate tissue biomarkers. Spectral-domain optical coherence tomography (SD-OCT) has become central to the clinical management of wet AMD, offering high-resolution visualization of retinal and subretinal structures. Key SD-OCT biomarkers include the presence and volume of intraretinal fluid (IRF), subretinal fluid (SRF), and pigment epithelial detachments (PEDs), all of which are strongly associated with active neovascularization and worse visual prognosis [110]. Quantitative changes in central retinal thickness and the dynamics of fluid compartments are routinely used to guide anti-VEGF therapy and assess treatment response [111]. Hyperreflective foci, seen as discrete hyperreflective dots within the neurosensory retina, have been linked to activated microglia or lipoprotein aggregates and are associated with chronic inflammation, photoreceptor degeneration, and progression to fibrosis [112]. Outer retinal tubulations and subretinal hyperreflective material are additional OCT features that reflect photoreceptor degeneration and chronicity of disease [113]. Beyond OCT, fundus autofluorescence and fluorescein angiography provide further insight into RPE health and the extent of neovascularization, although their use as quantitative biomarkers is more limited [114]. While direct molecular profiling of retinal tissue is rare in humans, transcriptomic and proteomic studies of surgically excised neovascularization membranes have revealed upregulation of angiogenic (VEGFA), inflammatory (CCL2, CXCL9), and extracellular matrix remodeling genes (THBS2, MMPs), mirroring findings in animal models [115] (Figure 2B).

#### 4.2.2. Serum and Plasma Biomarkers

Systemic biomarkers, particularly circulating miRNAs, have emerged as promising minimally invasive tools for wet AMD. Multiple studies have identified distinct miRNA signatures in whole blood, plasma, and serum of patients with neovascular AMD. For example, elevated levels of miR-27a-3p, miR-29b-3p, and miR-195-5p have been reported in whole blood, while miR-23a-3p, miR-30b, miR-191-5p, and miR-223-3p are upregulated in peripheral blood nuclear cells [69] (Figure 2B). Conversely, miR-16-5p, miR-17-3p, miR-150-5p, and miR-155-5p are downregulated in wet AMD compared to healthy controls [63]. In plasma, increased miR-16-5p, miR-30b, and miR-191-5p, along with decreased miR-23a-3p, have been observed, with several miRNAs (such as miR-26b-5p and miR-27b-3p) appearing specific to the neovascular form of the disease [116]. Serum analyses have revealed reduced levels of miR-34a-5p, miR-126-3p, miR-145-5p, and miR-205-5p, some of which target VEGFA, the principal driver of neovascularization [96] (Figure 2B). These miRNAs are functionally implicated in angiogenesis, inflammation, and extracellular matrix remodeling, and their conservation between human patients and animal models underscores their translational potential.

Beyond miRNAs, systemic markers of inflammation and oxidative stress have been investigated in wet AMD. Elevated plasma levels of CRP, complement activation fragments (e.g., C3a, C5a), and pro-inflammatory cytokines (e.g., IL-6 and TNF-α) have been associated with increased risk and activity of neovascular AMD, reflecting the role of systemic immune dysregulation in disease pathogenesis [117] (Figure 2B). Markers of oxidative stress, including malondialdehyde and advanced glycation end products, are also elevated in AMD patients, supporting the contribution of chronic oxidative injury to retinal degeneration [118].

#### 4.2.3. Directly Measurable Tear Fluid Biomarkers

Tear fluid has recently gained attention as a non-invasive source of biomarkers for wet AMD. Elevated concentrations of VEGF have been detected in the tears of patients with active neovascular AMD, paralleling findings from preclinical models [70]. Tear VEGF levels correlate with intraocular disease activity and may exhibit sex-specific differences, suggesting potential for personalized disease monitoring [70] (Figure 2B). The accessibility and repeatability of tear sampling make it particularly attractive for longitudinal studies and for assessing therapeutic response in clinical settings. Other tear-based markers, such as inflammatory cytokines and MMP, are under investigation, but their diagnostic and prognostic value in AMD remains to be fully established [70]. Recent data highlight tear fluid as a non-invasive source of candidate biomarkers for wet AMD, including VEGF, which shows elevated concentrations in exploratory patient studies. These studies remain at the discovery stage, and parallel findings in preclinical models further suggest a need for translational caution. The limited cohort sizes and varying collection protocols constrain generalizability and reproducibility. As such, rigorous verification and multicenter validation are essential, with protocols specifying standardized sampling, diurnal controls, and targeted assays (ELISA, PRM/MRM) tested on powered cohorts with registered statistical endpoints. The clinical significance of other tear-based markers (e.g., cytokines, MMPs) awaits thorough validation [70].

#### 4.2.4. Directly Measurable Aqueous and Vitreous Humor Biomarkers

Analysis of intraocular fluids, particularly aqueous and vitreous humor, has provided direct evidence of the molecular milieu associated with wet AMD. VEGF levels are consistently elevated in both aqueous and vitreous samples from patients with neovascular AMD, reflecting ongoing angiogenic activity and forming the basis for anti-VEGF therapeutic strategies [104]. In addition to VEGF, increased concentrations of fibrotic mediators such as thrombospondin 2 (THBS2), as well as various interleukins and matrix metalloproteinases (MMP-9), have been reported [119,120] (Figure 2B). These factors are involved in tissue remodeling, inflammation, and fibrosis, processes central to disease progression and vision loss. Proteomic analyses of vitreous samples have further identified dysregulation of proteins involved in complement activation, lipid metabolism, and ECM turnover, highlighting the complex molecular environment of the neovascular retina [121].

#### 4.2.5. Directly Measurable Microbiome Biomarkers

Emerging evidence suggests that the gut microbiome may influence AMD pathogenesis in humans, as in animal models. Distinct microbial signatures and metabolite profiles have been linked to AMD risk, although the clinical utility of stool-based biomarkers remains preliminary [48]. Notably, alterations in bacterial composition—such as increased *Lachnospiraceae* and decreased *Bacteroidetes*—have been reported in AMD patients compared with controls (Figure 2B) [122].

Collectively, these findings demonstrate that wet AMD in humans is associated with a diverse array of biomarkers detectable through advanced imaging, systemic and ocular fluid analysis, and genetic profiling. Many of these biomarkers are conserved across preclinical models and human disease, supporting their utility in translational research and personalized medicine for AMD [70].

- Understanding Confounding: Foundations and Assumptions of Mendelian Randomization

Several studies have reported altered gut microbiome profiles in AMD patients; however, these associations remain highly susceptible to confounding by diet, medication use, and lifestyle factors. Dietary intake can rapidly modulate microbiome composition, while commonly used medications—such as antibiotics, proton pump inhibitors, and statins, can substantially alter bacterial communities, potentially biasing cross-sectional observations [123]. Even when MR methods are applied, only suggestive causal relationships have been identified. For instance, taxa such as *Bacteroidales* S24.7 and *Ruminococcaceae* UCG011 have been linked to different AMD forms, yet these associations often fail replication or demonstrate modest effect sizes [124,125]. Furthermore, MR analyses rely on several critical assumptions, including the independence of genetic instruments, absence of pleiotropy, and stability of microbiome–exposure relationships over time—that may not hold in elderly AMD populations with multiple comorbidities. Collectively, these limitations suggest that while gut microbiome alterations represent promising biomarkers, they cannot yet be considered definitive causal indicators of AMD. In addition, microbiome shifts observed across cohorts may influence systemic inflammation and immune regulation, thereby impacting retinal health through the gut–retina axis. The consistent increase in *Lachnospiraceae* observed in laser-induced mouse models further underscores the translational relevance of these findings.

#### 4.2.6. Directly Measurable Genetic Biomarkers

Genetic association studies have identified several robust risk loci for AMD, most notably single nucleotide polymorphisms in the CFH gene and the ARMS2/HTRA1 locus (Figure 2B). These variants account for a substantial proportion of the heritable risk for AMD and are increasingly used to stratify patients in both clinical and research settings [6]. Additional genetic markers under investigation include variants in genes involved in lipid metabolism (e.g., APOE, LIPC), extracellular matrix regulation (e.g., TIMP3), and angiogenic signaling pathways (e.g., VEGFA), further expanding the landscape of genetic risk assessment in AMD [126,127,128] (Figure 2B). Polygenic risk scores integrating multiple loci are being developed to improve predictive accuracy for disease onset and progression [129].

## 5. Discussion: Translational Integration and Limits

Chemical mouse models of dry AMD, using agents like NaIO_3_, replicate human oxidative stress markers (8-OHdG, 4-HNE, CEP), pro-apoptotic proteins, inflammatory cytokines, complement activation, and MMP dysregulation, alongside gut microbiota alterations mirroring human disease [23,33,35,75,92,105,129]. Genetic models (e.g., Sod^−^/^−^, Sod2 knockdown, CFH variants) likewise show overlapping oxidative stress, inflammation, and complement dysregulation, with gene-microbiota interactions influencing systemic inflammation, though some systemic markers and late-stage features like GA are not fully replicated [38,39,49,54,116,129,130]. Laser-induced models of wet AMD reproduce key features of neovascularization, including VEGF elevation, inflammatory chemokines, extracellular matrix remodeling, and shared miRNA dysregulation, but differ anatomically (absence of macula), pathologically (acute vs. chronic progression), and in hallmark lesion formation (lack of drusen) [29,40,122,129,131,132,133]. While circulating markers such as complement fragments and interleukins have shown promise as indicators of disease activity in mouse models of AMD, few studies have quantitatively assessed their incremental predictive value beyond established genetic (e.g., CFH, ARMS2) or imaging biomarkers. Reported multivariable analyses suggest that complement components and interleukins may have independent associations even when adjusted for genetic risk. However, nested-model comparisons and corresponding performance metrics, such as ΔAUC and Net Reclassification Index (NRI), are not widely available in the current literature [134]. Therefore, while systemic proteomic and cytokine signatures provide valuable mechanistic insights, their potential to improve AMD risk stratification has yet to be systematically evaluated in preclinical and clinical settings.

Numerous candidate biomarkers for AMD have been identified across various biological domains—including oxidative stress, inflammation, complement activation, extracellular matrix remodeling, angiogenesis, and gut microbiota alterations. However, few studies have established standardized clinical thresholds. This lack of defined cut-points limits the translational utility of these biomarkers in clinical practice. To enhance their applicability, future research should focus on defining and validating preliminary cut-points using approaches such as Youden index optimization or clinically anchored thresholds [135]. These strategies can facilitate the development of diagnostic and prognostic tools that are both sensitive and specific, thereby improving early detection and personalized treatment strategies for AMD [136].

## 6. Conclusions: Toward Practical Biomarker Strategies for AMD

Reliable biomarkers are essential for improving AMD diagnosis, monitoring, and treatment, especially for dry AMD lacking cures. Biomarkers related to oxidative stress, inflammation, complement, matrix remodeling, angiogenesis, and gut microbiota show overlap between human AMD and preclinical models-genetic, chemical, and laser-induced (See Supplementary Figures S1 and S2). Each model reflects specific disease aspects, with chemical models offering reproducibility. Biomarkers span retina, fluids, and feces, highlighting translational value, but animal models face limitations in fluid analysis, macular anatomy, and chronicity. Mouse models share key features with neovascular AMD but differ anatomically and temporally, requiring cautious interpretation [69]. Preclinical findings need validation in humans for clinical relevance. Integrating experimental and patient data will aid in developing personalized, non-invasive biomarker strategies for AMD.

## Data Availability

No new data were created or analyzed in this study.

## Supplementary Materials

The following supporting information can be downloaded at https://www.mdpi.com/article/10.3390/biom15111571/s1: Detailed quantitative and qualitative biomarker metrics for AMD are summarized in Supplementary Figure S1; Overview of major biomarkers identified in preclinical mouse models and human AMD are summarized in Supplementary Figure S2.

## Abbreviations

The following abbreviations are used in this manuscript:

AMD	Age-related Macular Degeneration
GA	Geographic Atrophy
RPE	Retinal Pigment Epithelium
VEGF	Vascular Endothelial Growth Factor
FDA	Food and Drug Administration
CFH	Complement Factor H
ARMS2	Age-Related Maculopathy Susceptibility 2
HTRA1	HtrA serine peptidase 1
C3	Complement component 3
miRNAs	microRNAs
MNU	N-methyl-N-nitrosourea
ROS	Reactive oxygen species
BAX	Bcl-2-associated X protein
MCP	Monocyte Chemoattractant Protein
GFAP	Glial Fibrillary Acidic Protein
MMP	Matrix metalloproteinase
TIMP-3	Tissue Inhibitor of Metalloproteinases-3
ECM	Extracellular matrix
ANGP	Angiopoietin
SAA	Serum Amyloid A
CCL2	Chemokine ligand 2
EPO	Erythropoietin
SCFAs	Short-Chain Fatty Acids
LPS	Lipopolysaccharide
AAE	Aronia Anthocyanidin Extract
C-X3-C	Motif Chemokine Receptor 1
VLDRL	Very low-density lipoprotein receptor
8-OHDG	8-hydroxy-2′-deoxyguanosine
IRAK-M	interleukin-1 receptor–associated kinase M
AAV	Adeno-Associated Virus
BrM	Bruch’s membrane
SNP	Single Nucleotide Polymorphism
MNV	Macular Neovascularization
THBS2	Thrombospondin 2
DHA	Docosahexaenoic Acid
CEP	Carboxyethyl Pyrrole
MDA	Malondialdehyde
CRP	C-reactive protein
SMOC2	Secreted Modular Calcium-binding protein 2
SD-OCT	Spectral-Domain Optical Coherence Tomography
IRF	Intraretinal Fluid
SRF	Subretinal Fluid
PEDs	Pigment Epithelial Detachments

## References

[1] Wong W.L., Su X., Li X., Cheung C.M.G., Klein R., Cheng C.-Y., Wong T.Y. (2014). Global prevalence of age-related macular degeneration and disease burden projection for 2020 and 2040: A systematic review and meta-analysis. Lancet Glob. Health.

[2] Curcio C.A., Kar D., Owsley C., Sloan K.R., Ach T. (2024). Age-Related Macular Degeneration, a Mathematically Tractable Disease. Investig. Ophthalmol. Vis. Sci.

[3] Choudhary M., Malek G. (2019). A Review of Pathogenic Drivers of Age-Related Macular Degeneration, Beyond Complement, and Potential Endpoints to Test Therapeutic Interventions in Preclinical Studies. Adv. Exp. Med. Biol..

[4] Wong J.H.C., Ma J.Y.W., Jobling A.I., Brandli A., Greferath U., Fletcher E.L., Vessey K.A. (2022). Exploring the pathogenesis of age-related macular degeneration: A review of the interplay between retinal pigment epithelium dysfunction and the innate immune system. Front. Neurosci..

[5] Bowes Rickman C., Farsiu S., Toth C.A., Klingeborn M. (2013). Dry Age-Related Macular Degeneration: Mechanisms, Therapeutic Targets, and Imaging. Investig. Ophthalmol. Vis. Sci..

[6] Fritsche L.G., Igl W., Cooke Bailey J.N., Grassmann F., Sengupta S., Bragg-Gresham J.L., Burdon K.P., Hebbring S.J., Wen C., Gorski M. (2016). A large genome-wide association study of age-related macular degeneration highlights contributions of rare and common variants. Nat. Genet..

[7] Hageman G.S., Gehrs K., Lejnine S., Bansal A.T., DeAngelis M.M., Guymer R.H., Baird P.N., Allikmets R., Deciu C., Oeth P. (2011). Clinical validation of a genetic model to estimate the risk of developing choroidal neovascular age-related macular degeneration. Hum. Genom..

[8] Lad E.M., Finger R.P., Guymer R. (2023). Biomarkers for the Progression of Intermediate Age-Related Macular Degeneration. Ophthalmol. Ther..

[9] Li S., Qiu Y., Li Y., Wu J., Yin N., Ren J., Shao M., Yu J., Song Y., Sun X. (2024). Serum metabolite biomarkers for the early diagnosis and monitoring of age-related macular degeneration. J. Adv. Res..

[10] Borrelli E., Serafino S., Ricardi F., Coletto A., Neri G., Olivieri C., Ulla L., Foti C., Marolo P., Toro M.D. (2024). Deep Learning in Neovascular Age-Related Macular Degeneration. Medicina.

[11] Exploring the Role of Exosomal miRNA-146a-5p in Sodium Iodate-Induced Retinal Pigment Epithelial Dysfunction | IOVS | ARVO Journals. https://iovs.arvojournals.org/article.aspx?articleid=2795919.

[12] Chen Y., Liu S., Hu D., Xing Y., Shen Y. (2014). N -methyl- N -nitrosourea-induced retinal degeneration in mice. Exp. Eye Res..

[13] Zhao J., Liao Y., Chen J., Dong X., Gao Z., Zhang H., Wu X., Liu Z., Wu Y. (2017). Aberrant Buildup of All-Trans-Retinal Dimer, a Nonpyridinium Bisretinoid Lipofuscin Fluorophore, Contributes to the Degeneration of the Retinal Pigment Epithelium. Investig. Ophthalmol. Vis. Sci..

[14] Zhang J., Bai Y., Huang L., Qi Y., Zhang Q., Li S., Wu Y., Li X. (2015). Protective effect of autophagy on human retinal pigment epithelial cells against lipofuscin fluorophore A2E: Implications for age-related macular degeneration. Cell Death Dis..

[15] Chen X., Tzekov R., Su M., Zhu Y., Han A., Li W. (2023). Hydrogen peroxide-induced oxidative damage and protective role of peroxiredoxin 6 protein via EGFR/ERK signaling pathway in RPE cells. Front. Aging Neurosci..

[16] Kaczara P., Sarna T., Burke J.M. (2010). Dynamics of H2O2 Availability to ARPE-19 Cultures in Models of Oxidative Stress. Free Radic. Biol. Med..

[17] You L., Zhao W., Li X., Yang C., Guo P. (2025). Tyrosol protects RPE cells from H_2_O_2_-induced oxidative damage in vitro and in vivo through activation of the Nrf2/HO-1 pathway. Eur. J. Pharmacol..

[18] Hara A., Niwa M., Aoki H., Kumada M., Kunisada T., Oyama T., Yamamoto T., Kozawa O., Mori H. (2006). A new model of retinal photoreceptor cell degeneration induced by a chemical hypoxia-mimicking agent, cobalt chloride. Brain Res..

[19] Muñoz-Sánchez J., Chánez-Cárdenas M.E. (2019). The use of cobalt chloride as a chemical hypoxia model. J. Appl. Toxicol..

[20] Ramkumar H.L., Zhang J., Chan C.-C. (2010). Retinal Ultrastructure of Murine Models of Dry Age-related Macular Degeneration (AMD). Prog. Retin. Eye Res..

[21] Montezuma S.R., Sobrin L., Seddon J.M. (2007). Review of Genetics in Age Related Macular Degeneration. Semin. Ophthalmol..

[22] Hanus J., Anderson C., Wang S. (2015). RPE Necroptosis in Response to Oxidative Stress and in AMD. Ageing Res. Rev..

[23] Enzbrenner A., Zulliger R., Biber J., Pousa A.M.Q., Schäfer N., Stucki C., Giroud N., Berrera M., Kortvely E., Schmucki R. (2021). Sodium Iodate-Induced Degeneration Results in Local Complement Changes and Inflammatory Processes in Murine Retina. Int. J. Mol. Sci..

[24] Liu R.T., Gao J., Cao S., Sandhu N., Cui J.Z., Chou C.L., Fang E., Matsubara J.A. (2013). Inflammatory Mediators Induced by Amyloid-Beta in the Retina and RPE In Vivo: Implications for Inflammasome Activation in Age-Related Macular Degeneration. Investig. Ophthalmol. Vis. Sci..

[25] Cervellati F., Cervellati C., Romani A., Cremonini E., Sticozzi C., Belmonte G., Pessina F., Valacchi G. (2014). Hypoxia induces cell damage via oxidative stress in retinal epithelial cells. Free Radic. Res..

[26] Lazzara F., Trotta M.C., Platania C.B.M., D’Amico M., Petrillo F., Galdiero M., Gesualdo C., Rossi S., Drago F., Bucolo C. (2020). Stabilization of HIF-1α in Human Retinal Endothelial Cells Modulates Expression of miRNAs and Proangiogenic Growth Factors. Front. Pharmacol..

[27] Zhang C.-J., Xiang L., Chen X.-J., Wang X.-Y., Wu K.-C., Zhang B.-W., Chen D.-F., Jin G.-H., Zhang H., Chen Y.-C. (2020). Ablation of Mature miR-183 Leads to Retinal Dysfunction in Mice. Investig. Ophthalmol. Vis. Sci..

[28] Fasanaro P., D’Alessandra Y., Di Stefano V., Melchionna R., Romani S., Pompilio G., Capogrossi M.C., Martelli F. (2008). MicroRNA-210 modulates endothelial cell response to hypoxia and inhibits the receptor tyrosine kinase ligand Ephrin-A3. J. Biol. Chem..

[29] Shu Y., Li Z., Zong T., Mu T., Zhou H., Yang Q., Wu M., Liu Y., Xie T., Tan C. (2025). MiR-21-5p promotes RPE cell necroptosis by targeting Peli1 in a rat model of AMD. In. Vitro Cell Dev. Biol. Anim..

[30] Tong Y., Wu Y., Ma J., Ikeda M., Ide T., Griffin C.T., Ding X.-Q., Wang S. (2023). Comparative mechanistic study of RPE cell death induced by different oxidative stresses. Redox Biol..

[31] Yang H.-J., Hu R., Sun H., Chen B., Li X., Chen J.-B. (2019). 4-HNE induces proinflammatory cytokines of human retinal pigment epithelial cells by promoting extracellular efflux of HSP70. Exp. Eye Res..

[32] Penn J.S., Madan A., Caldwell R.B., Bartoli M., Caldwell R.W., Hartnett M.E. (2008). Vascular Endothelial Growth Factor in Eye Disease. Prog. Retin. Eye Res..

[33] Spilsbury K., Garrett K.L., Shen W.Y., Constable I.J., Rakoczy P.E. (2000). Overexpression of vascular endothelial growth factor (VEGF) in the retinal pigment epithelium leads to the development of choroidal neovascularization. Am. J. Pathol..

[34] Luo L., Uehara H., Zhang X., Das S.K., Olsen T., Holt D., Simonis J.M., Jackman K., Singh N., Miya T.R. (2013). Photoreceptor avascular privilege is shielded by soluble VEGF receptor-1. eLife.

[35] Hanus J., Anderson C., Sarraf D., Ma J., Wang S. (2016). Retinal pigment epithelial cell necroptosis in response to sodium iodate. Cell Death Discov..

[36] Gu Y., Liu W., Liu G., Li X., Lu P. (2021). Assessing the protective effects of cryptotanshinone on CoCl_2_-induced hypoxia in RPE cells. Mol. Med. Rep..

[37] Xing Y., Liang S., Zhang L., Ni H., Zhang X., Wang J., Yang L., Song S., Li H.-H., Jia C. (2023). Combination of Lactobacillus fermentum NS9 and aronia anthocyanidin extract alleviates sodium iodate-induced retina degeneration. Sci. Rep..

[38] Nguyen Y., Rudd Zhong Manis J., Ronczkowski N.M., Bui T., Oxenrider A., Jadeja R.N., Thounaojam M.C. (2024). Unveiling the gut-eye axis: How microbial metabolites influence ocular health and disease. Front. Med..

[39] Raoul W., Auvynet C., Camelo S., Guillonneau X., Feumi C., Combadière C., Sennlaub F. (2010). CCL2/CCR2 and CX3CL1/CX3CR1 chemokine axes and their possible involvement in age-related macular degeneration. J. Neuroinflammation.

[40] Vessey K.A., Greferath U., Jobling A.I., Phipps J.A., Ho T., Waugh M., Fletcher E.L. (2012). Ccl2/Cx3cr1 knockout mice have inner retinal dysfunction but are not an accelerated model of AMD. Investig. Ophthalmol. Vis. Sci..

[41] Ross R.J., Zhou M., Shen D., Fariss R.N., Ding X., Bojanowski C.M., Tuo J., Chan C.-C. (2008). Immunological protein expression profile in Ccl2/Cx3cr1 deficient mice with lesions similar to age-related macular degeneration. Exp. Eye Res..

[42] Imamura Y., Noda S., Hashizume K., Shinoda K., Yamaguchi M., Uchiyama S., Shimizu T., Mizushima Y., Shirasawa T., Tsubota K. (2006). Drusen, choroidal neovascularization, and retinal pigment epithelium dysfunction in SOD1-deficient mice: A model of age-related macular degeneration. Proc. Natl. Acad. Sci. USA.

[43] Hashizume K., Hirasawa M., Imamura Y., Noda S., Shimizu T., Shinoda K., Kurihara T., Noda K., Ozawa Y., Ishida S. (2008). Retinal Dysfunction and Progressive Retinal Cell Death in SOD1-Deficient Mice. Am. J. Pathol..

[44] Zhu Y., Aredo B., Chen B., Zhao C.X., He Y.-G., Ufret-Vincenty R.L. (2019). Mice with a Combined Deficiency of Superoxide Dismutase 1 (Sod1), DJ-1 (Park7), and Parkin (Prkn) Develop Spontaneous Retinal Degeneration with Aging. Investig. Ophthalmol. Vis. Sci..

[45] Lin M.K., Yang J., Hsu C.W., Gore A., Bassuk A.G., Brown L.M., Colligan R., Sengillo J.D., Mahajan V.B., Tsang S.H. (2018). HTRA1, an age-related macular degeneration protease, processes extracellular matrix proteins EFEMP1 and TSP1. Aging Cell.

[46] Vierkotten S., Muether P.S., Fauser S. (2011). Overexpression of HTRA1 leads to ultrastructural changes in the elastic layer of Bruch’s membrane via cleavage of extracellular matrix components. PLoS ONE.

[47] Chen C.-Y., Melo E., Jakob P., Friedlein A., Elsässer B., Goettig P., Kueppers V., Delobel F., Stucki C., Dunkley T. (2018). N-Terminomics identifies HtrA1 cleavage of thrombospondin-1 with generation of a proangiogenic fragment in the polarized retinal pigment epithelial cell model of age-related macular degeneration. Matrix Biol..

[48] Zinkernagel M.S., Zysset-Burri D.C., Keller I., Berger L.E., Leichtle A.B., Largiadèr C.R., Fiedler G.M., Wolf S. (2017). Association of the Intestinal Microbiome with the Development of Neovascular Age-Related Macular Degeneration. Sci. Rep..

[49] Bringer M.-A., Gabrielle P.-H., Bron A.M., Creuzot-Garcher C., Acar N. (2022). The gut microbiota in retinal diseases. Exp. Eye Res..

[50] Pennesi M.E., Neuringer M., Courtney R.J. (2012). Animal models of age related macular degeneration. Mol. Aspects Med..

[51] Badia A., Salas A., Duarri A., Ferreira-de-Souza B., Zapata M.Á., Fontrodona L., García-Arumí J. (2021). Transcriptomics analysis of Ccl2/Cx3cr1/Crb1rd8 deficient mice provides new insights into the pathophysiology of progressive retinal degeneration. Exp. Eye Res..

[52] Liu J., Copland D.A., Clare A.J., Gorski M., Richards B.T., Scott L., Theodoropoulou S., Greferath U., Cox K., Shi G. (2024). Replenishing IRAK-M expression in retinal pigment epithelium attenuates outer retinal degeneration. Sci. Transl. Med..

[53] Liu J., Copland D.A., Clare A.J., Gorski M., Richards B.T., Scott L., Theodoropoulou S., Greferath U., Cox K., Bell O.H. (2023). Replenishing Age-Related Decline of IRAK-M Expression in Retinal Pigment Epithelium Attenuates Outer Retinal Degeneration. bioRxiv.

[54] Jones A., Kumar S., Zhang N., Tong Z., Yang J.-H., Watt C., Anderson J., Amrita, Fillerup H., McCloskey M. (2011). Increased expression of multifunctional serine protease, HTRA1, in retinal pigment epithelium induces polypoidal choroidal vasculopathy in mice. Proc. Natl. Acad. Sci. USA.

[55] Zysset-Burri D.C., Keller I., Berger L.E., Largiadèr C.R., Wittwer M., Wolf S., Zinkernagel M.S. (2020). Associations of the intestinal microbiome with the complement system in neovascular age-related macular degeneration. NPJ Genom. Med..

[56] Rowan S., Taylor A. (2018). Gut microbiota modify risk for dietary glycemia-induced age-related macular degeneration. Gut Microbes.

[57] Shah R.S., Soetikno B.T., Lajko M., Fawzi A.A. (2015). A Mouse Model for Laser-induced Choroidal Neovascularization. J. Vis. Exp..

[58] Jimenez A.I., Bernabeu-Zornoza A., Esteve J.J., Gombau A., Matei A., Ramos F., Martínez-Navarrete G., Fernandez E., Sylentis T. (2023). Optimization and characterization of an improved laser-induced choroidal neovascularization animal model for the study of retinal diseases. Investig. Ophthalmol. Vis. Sci..

[59] Gong Y., Li J., Sun Y., Fu Z., Liu C.-H., Evans L., Tian K., Saba N., Fredrick T., Morss P. (2015). Optimization of an Image-Guided Laser-Induced Choroidal Neovascularization Model in Mice. PLoS ONE.

[60] Salas A., Badia A., Fontrodona L., Zapata M., García-Arumí J., Duarri A. (2023). Neovascular Progression and Retinal Dysfunction in the Laser-Induced Choroidal Neovascularization Mouse Model. Biomedicines.

[61] Liu Y.-S., Pan J.-Q., Pan X.-B., Kong F.-S., Zhang J.-Q., Wei Z.-Y., Xu Z.-H., Rao J.-H., Wang J.-H., Chen J.-H. (2024). Comparative Analysis of Molecular Landscape in Mouse Models and Patients Reveals Conserved Inflammation Pathways in Age-Related Macular Degeneration. Investig. Ophthalmol. Vis. Sci..

[62] Wolf J., Schlecht A., Rosmus D.-D., Boneva S., Agostini H., Schlunck G., Wieghofer P., Lange C. (2022). Comparative transcriptome analysis of human and murine choroidal neovascularization identifies fibroblast growth factor inducible-14 as phylogenetically conserved mediator of neovascular age-related macular degeneration. Biochim. Biophys. Acta Mol. Basis Dis..

[63] Toma H.S., Barnett J.M., Penn J.S., Kim S.J. (2010). Improved assessment of laser-induced choroidal neovascularization. Microvasc. Res..

[64] Iwanishi H., Yamanaka O., Sumioka T., Yasuda S., Miyajima M., Saika S. (2022). Delayed regression of laser-induced choroidal neovascularization in TNFα-null mice. J. Cell. Mol. Med..

[65] Lambert V., Lecomte J., Hansen S., Blacher S., Gonzalez M.-L.A., Struman I., Sounni N.E., Rozet E., de Tullio P., Foidart J.M. (2013). Laser-induced choroidal neovascularization model to study age-related macular degeneration in mice. Nat. Protoc..

[66] Hu Y., Qi S., Zhuang H., Zhuo Q., Liang Y., Kong H., Zhao C., Zhang S. (2023). Proteotranscriptomic analyses reveal distinct interferon-beta signaling pathways and therapeutic targets in choroidal neovascularization. Front. Immunol..

[67] Apte R.S., Richter J., Herndon J., Ferguson T.A. (2006). Macrophages Inhibit Neovascularization in a Murine Model of Age-Related Macular Degeneration. PLoS Med..

[68] Brandli A., Khong F.L., Kong R.C.K., Kelly D.J., Fletcher E.L. (2022). Transcriptomic analysis of choroidal neovascularization reveals dysregulation of immune and fibrosis pathways that are attenuated by a novel anti-fibrotic treatment. Sci. Rep..

[69] Kiel C., Berber P., Karlstetter M., Aslanidis A., Strunz T., Langmann T., Grassmann F., Weber B.H.F. (2020). A Circulating MicroRNA Profile in a Laser-Induced Mouse Model of Choroidal Neovascularization. Int. J. Mol. Sci..

[70] Moshtaghion S.M.M., Locri F., Reyes A.P., Plastino F., Kvanta A., Morillo-Sanchez M.J., Rodríguez-de-la-Rúa E., Gutierrez-Sanchez E., Montero-Sánchez A., Lucena-Padros H. (2025). VEGF in Tears as a Biomarker for Exudative Age-Related Macular Degeneration: Molecular Dynamics in a Mouse Model and Human Samples. Int. J. Mol. Sci..

[71] Dos Santos F.M., Ciordia S., Mesquita J., de Sousa J.P.C., Paradela A., Tomaz C.T., Passarinha L.A.P. (2022). Vitreous humor proteome: Unraveling the molecular mechanisms underlying proliferative and neovascular vitreoretinal diseases. Cell Mol. Life Sci..

[72] Oca A.I., Pérez-Sala Á., Pariente A., Ochoa R., Velilla S., Peláez R., Larráyoz I.M. (2021). Predictive Biomarkers of Age-Related Macular Degeneration Response to Anti-VEGF Treatment. J. Pers. Med..

[73] Ratnapriya R., Chew E.Y. (2013). Age-related macular degeneration-clinical review and genetics update. Clin. Genet..

[74] Andriessen E.M., Wilson A.M., Mawambo G., Dejda A., Miloudi K., Sennlaub F., Sapieha P. (2016). Gut microbiota influences pathological angiogenesis in obesity-driven choroidal neovascularization. EMBO Mol. Med..

[75] Zhang H., Mo Y. (2023). The gut-retina axis: A new perspective in the prevention and treatment of diabetic retinopathy. Front. Endocrinol..

[76] Bakri S.J., Bektas M., Sharp D., Luo R., Sarda S.P., Khan S. (2023). Geographic atrophy: Mechanism of disease, pathophysiology, and role of the complement system. J. Manag. Care Spec. Pharm..

[77] Lau L.-I., Liu C.J., Wei Y.-H. (2010). Increase of 8-hydroxy-2’-deoxyguanosine in aqueous humor of patients with exudative age-related macular degeneration. Investig. Ophthalmol. Vis. Sci..

[78] Shen J.K., Dong A., Hackett S.F., Bell W.R., Green W.R., Campochiaro P.A. (2007). Oxidative damage in age-related macular degeneration. Histol. Histopathol..

[79] Crabb J.W., Miyagi M., Gu X., Shadrach K., West K.A., Sakaguchi H., Kamei M., Hasan A., Yan L., Rayborn M.E. (2002). Drusen proteome analysis: An approach to the etiology of age-related macular degeneration. Proc. Natl. Acad. Sci. USA.

[80] Lu L., Gu X., Hong L., Laird J., Jaffe K., Choi J., Crabb J., Salomon R.G. (2009). Synthesis and structural characterization of carboxyethylpyrrole-modified proteins: Mediators of age-related macular degeneration. Bioorg. Med. Chem..

[81] Gu X., Meer S.G., Miyagi M., Rayborn M.E., Hollyfield J.G., Crabb J.W., Salomon R.G. (2003). Carboxyethylpyrrole protein adducts and autoantibodies, biomarkers for age-related macular degeneration. J. Biol. Chem..

[82] Renganathan K., Gu J., Rayborn M.E., Crabb J.S., Salomon R.G., Collier R.J., Kapin M.A., Romano C., Hollyfield J.G., Crabb J.W. (2013). CEP Biomarkers as Potential Tools for Monitoring Therapeutics. PLoS ONE.

[83] Ethen C.M., Reilly C., Feng X., Olsen T.W., Ferrington D.A. (2007). Age-related macular degeneration and retinal protein modification by 4-hydroxy-2-nonenal. Investig. Ophthalmol. Vis. Sci..

[84] Gu J., Pauer G.J.T., Yue X., Narendra U., Sturgill G.M., Bena J., Gu X., Peachey N.S., Salomon R.G., Hagstrom S.A. (2010). Proteomic and genomic biomarkers for age-related macular degeneration. Adv. Exp. Med. Biol..

[85] Totan Y., Yağci R., Bardak Y., Ozyurt H., Kendir F., Yilmaz G., Sahin S., Sahin Tiğ U. (2009). Oxidative macromolecular damage in age-related macular degeneration. Curr. Eye Res..

[86] Nita M., Strzałka-Mrozik B., Grzybowski A., Mazurek U., Romaniuk W. (2014). Age-related macular degeneration and changes in the extracellular matrix. Med. Sci. Monit..

[87] Hussain A.A., Lee Y., Zhang J.-J., Marshall J. (2011). Disturbed Matrix Metalloproteinase Activity of Bruch’s Membrane in Age-Related Macular Degeneration. Investig. Ophthalmol. Vis. Sci..

[88] Abokyi S., To C.-H., Lam T.T., Tse D.Y. (2020). Central Role of Oxidative Stress in Age-Related Macular Degeneration: Evidence from a Review of the Molecular Mechanisms and Animal Models. Oxid. Med. Cell Longev..

[89] Chen W., Li Z., Zhou X., Li C., Lin Y. (2025). Identification of Biomarkers for Oxidative Stress in Age-Related Macular Degeneration: Combining Transcriptomics and Mendelian Randomization Analysis. Transl. Vis. Sci. Technol..

[90] Hageman G.S., Anderson D.H., Johnson L.V., Hancox L.S., Taiber A.J., Hardisty L.I., Hageman J.L., Stockman H.A., Borchardt J.D., Gehrs K.M. (2005). A common haplotype in the complement regulatory gene factor H (HF1/CFH) predisposes individuals to age-related macular degeneration. Proc. Natl. Acad. Sci. USA.

[91] Reynolds R., Hartnett M.E., Atkinson J.P., Giclas P.C., Rosner B., Seddon J.M. (2009). Plasma complement components and activation fragments: Associations with age-related macular degeneration genotypes and phenotypes. Investig. Ophthalmol. Vis. Sci..

[92] Ildefonso C.J., Biswal M.R., Ahmed C.M., Lewin A.S. (2016). The NLRP3 Inflammasome and its Role in Age-Related Macular Degeneration. Adv. Exp. Med. Biol..

[93] Szaflik J.P., Janik-Papis K., Synowiec E., Ksiazek D., Zaras M., Wozniak K., Szaflik J., Blasiak J. (2009). DNA damage and repair in age-related macular degeneration. Mutat. Res..

[94] Martinez B., Peplow P.V. (2020). MicroRNAs as diagnostic and prognostic biomarkers of age-related macular degeneration: Advances and limitations. Neural Regen. Res..

[95] Berber P., Grassmann F., Kiel C., Weber B.H.F. (2017). An Eye on Age-Related Macular Degeneration: The Role of MicroRNAs in Disease Pathology. Mol. Diagn. Ther..

[96] Cruz-Aguilar M., Groman-Lupa S., Jiménez-Martínez M.C. (2023). MicroRNAs as potential biomarkers and therapeutic targets in age-related macular degeneration. Front. Ophthalmol..

[97] Zafrilla P., Losada M., Perez A., Caravaca G., Mulero J. (2013). Biomarkers of oxidative stress in patients with wet age related macular degeneration. J. Nutr. Health Aging.

[98] Yuan L.-Y., Su W.-M., Li L.-P., Tian X.-F., Zheng X.-L., Yuan X.-Y. (2025). Causal role of oxidative stress in age-related macular degeneration: A bidirectional Mendelian randomization study. Int. J. Ophthalmol..

[99] Winiarczyk M., Kaarniranta K., Winiarczyk S., Adaszek Ł., Winiarczyk D., Mackiewicz J. (2018). Tear film proteome in age-related macular degeneration. Graefes Arch. Clin. Exp. Ophthalmol..

[100] Kaarniranta K., Tokarz P., Koskela A., Paterno J., Blasiak J. (2017). Autophagy regulates death of retinal pigment epithelium cells in age-related macular degeneration. Cell Biol. Toxicol..

[101] Zhou L., Beuerman R.W. (2017). The power of tears: How tear proteomics research could revolutionize the clinic. Expert Rev. Proteom..

[102] Zhou L., Beuerman R.W., Chan C.M., Zhao S.Z., Li X.R., Yang H., Tong L., Liu S., Stern M.E., Tan D. (2009). Identification of tear fluid biomarkers in dry eye syndrome using iTRAQ quantitative proteomics. J. Proteome Res..

[103] Baek J.-H., Lim D., Park K.H., Chae J.-B., Jang H., Lee J., Chung H. (2018). Quantitative proteomic analysis of aqueous humor from patients with drusen and reticular pseudodrusen in age-related macular degeneration. BMC Ophthalmol..

[104] Rinsky B., Beykin G., Grunin M., Amer R., Khateb S., Tiosano L., Almeida D., Hagbi-Levi S., Elbaz-Hayoun S., Chowers I. (2021). Analysis of the Aqueous Humor Proteome in Patients with Age-Related Macular Degeneration. Investig. Ophthalmol. Vis. Sci..

[105] Yoon E.G., Nam K.T., Choi M., Choi K.-E., Yun C. (2025). Aqueous Humor Levels of Vascular Endothelial Growth Factor in Patients With Dry Age-Related Macular Degeneration and Subretinal Drusenoid Deposits. Investig. Ophthalmol. Vis. Sci..

[106] García-Quintanilla L., Rodríguez-Martínez L., Bandín-Vilar E., Gil-Martínez M., González-Barcia M., Mondelo-García C., Fernández-Ferreiro A., Mateos J. (2022). Recent Advances in Proteomics-Based Approaches to Studying Age-Related Macular Degeneration: A Systematic Review. Int. J. Mol. Sci..

[107] Huang H.-Y., Wang J., Qin B., Tan Y. (2024). Investigating the causal link between gut microbiota and dry age-related macular degeneration: A bidirectional Mendelian randomization study. Int. J. Ophthalmol..

[108] Xiao J., Zhang J.Y., Luo W., He P.C., Skondra D. (2023). The Emerging Role of Gut Microbiota in Age-Related Macular Degeneration. Am. J. Pathol..

[109] Ambati J., Fowler B.J. (2012). Mechanisms of age-related macular degeneration. Neuron.

[110] Wong D.T., Berger A.R., Bourgault S., Chen J., Colleaux K., Cruess A.F., Dookeran R.I., Gauthier D., Hurley B., Kapusta M.A. (2021). Imaging Biomarkers and Their Impact on Therapeutic Decision-Making in the Management of Neovascular Age-Related Macular Degeneration. Ophthalmologica.

[111] Kaiser P.K., Wykoff C.C., Singh R.P., Khanani A.M., Do D.V., Patel H., Patel N. (2021). Retinal fluid and thickness as measures of disease activity in neovascular age-related macular degeneration. Retina.

[112] Rispoli M., Cennamo G., Antonio L.D., Lupidi M., Parravano M., Pellegrini M., Veritti D., Vujosevic S., Savastano M.C. (2023). Practical guidance for imaging biomarkers in exudative age-related macular degeneration. Surv. Ophthalmol..

[113] Litts K.M., Ach T., Hammack K.M., Sloan K.R., Zhang Y., Freund K.B., Curcio C.A. (2016). Quantitative Analysis of Outer Retinal Tubulation in Age-Related Macular Degeneration from Spectral-Domain Optical Coherence Tomography and Histology. Investig. Ophthalmol. Vis. Sci..

[114] Hammer M., Oertel J., Alderzy H., Tarhan M., Meller D., Curcio C.A. (2025). Fundus autofluorescence intensity, lifetime, and spectral imaging in age-related macular degeneration. Exp. Eye Res..

[115] Schlecht A., Boneva S., Gruber M., Zhang P., Horres R., Bucher F., Auw-Haedrich C., Hansen L., Stahl A., Hilgendorf I. (2020). Transcriptomic Characterization of Human Choroidal Neovascular Membranes Identifies Calprotectin as a Novel Biomarker for Patients with Age-Related Macular Degeneration. Am. J. Pathol..

[116] ElShelmani H., Brennan I., Kelly D.J., Keegan D. (2021). Differential Circulating MicroRNA Expression in Age-Related Macular Degeneration. Int. J. Mol. Sci..

[117] Kauppinen A., Paterno J.J., Blasiak J., Salminen A., Kaarniranta K. (2016). Inflammation and its role in age-related macular degeneration. Cell. Mol. Life Sci..

[118] Beatty S., Koh H., Phil M., Henson D., Boulton M. (2000). The role of oxidative stress in the pathogenesis of age-related macular degeneration. Surv. Ophthalmol..

[119] Caban M., Owczarek K., Lewandowska U. (2022). The Role of Metalloproteinases and Their Tissue Inhibitors on Ocular Diseases: Focusing on Potential Mechanisms. Int. J. Mol. Sci..

[120] Wilson S., Siebourg-Polster J., Titz B., Jiang Z., Bartolo F., Lavergne V., Gayán J., Garweg J.G., Fauser S., Dieckmann A. (2023). Correlation of Aqueous, Vitreous, and Serum Protein Levels in Patients with Retinal Diseases. Transl. Vis. Sci. Technol..

[121] Nobl M., Reich M., Dacheva I., Siwy J., Mullen W., Schanstra J.P., Choi C.Y., Kopitz J., Kretz F.T.A., Auffarth G.U. (2016). Proteomics of vitreous in neovascular age-related macular degeneration. Exp. Eye Res..

[122] Schiavone N., Isoldi G., Calcagno S., Rovida E., Antiga E., De Almeida C.V., Lulli M. (2025). Exploring the Gut Microbiota–Retina Axis: Implications for Health and Disease. Microorganisms.

[123] Li C., Lu P. (2023). Association of Gut Microbiota with Age-Related Macular Degeneration and Glaucoma: A Bidirectional Mendelian Randomization Study. Nutrients.

[124] Liu K., Zou J., Yuan R., Fan H., Hu H., Cheng Y., Liu J., Zou H., You Z. (2023). Exploring the Effect of the Gut Microbiome on the Risk of Age-Related Macular Degeneration from the Perspective of Causality. Investig. Ophthalmol. Vis. Sci..

[125] Wei P., Gao S., Han G. (2025). Evidence for Genetic Causal Association Between the Gut Microbiome, Derived Metabolites, and Age-Related Macular Degeneration: A Mediation Mendelian Randomization Analysis. Biomedicines.

[126] Fritsche L.G., Chen W., Schu M., Yaspan B.L., Yu Y., Thorleifsson G., Zack D.J., Arakawa S., Cipriani V., Ripke S. (2013). Seven new loci associated with age-related macular degeneration. Nat. Genet..

[127] Kaur I., Rathi S., Chakrabarti S. (2010). Variations in TIMP3 are associated with age-related macular degeneration. Proc. Natl. Acad. Sci. USA.

[128] Churchill A.J., Carter J.G., Lovell H.C., Ramsden C., Turner S.J., Yeung A., Escardo J., Atan D. (2006). VEGF polymorphisms are associated with neovascular age-related macular degeneration. Hum. Mol. Genet..

[129] Yu C., Robman L., He W., Woods R.L., Phuong Thao L.T., Wolfe R., Phung J., Makeyeva G.A., Hodgson L.A.B., McNeil J.J. (2024). Predictive Performance of an Updated Polygenic Risk Score for Age-Related Macular Degeneration. Ophthalmology.

[130] Cai X., Seal S., McGinnis J.F. (2014). Sustained inhibition of neovascularization in vldlr-/- mice following intravitreal injection of cerium oxide nanoparticles and the role of the ASK1-P38/JNK-NF-κB pathway. Biomaterials.

[131] Sennlaub F., Auvynet C., Calippe B., Lavalette S., Poupel L., Hu S.J., Dominguez E., Camelo S., Levy O., Guyon E. (2013). CCR2(+) monocytes infiltrate atrophic lesions in age-related macular disease and mediate photoreceptor degeneration in experimental subretinal inflammation in Cx3cr1 deficient mice. EMBO Mol. Med..

[132] Choi Y.J., Lim D., Byeon S.H., Shin E.-C., Chung H. (2022). Chemokine Receptor Profiles of T Cells in Patients with Age-Related Macular Degeneration. Yonsei Med. J..

[133] Merkle C.W., Augustin M., Harper D.J., Glösmann M., Baumann B. (2022). Degeneration of Melanin-Containing Structures Observed Longitudinally in the Eyes of SOD1-/- Mice Using Intensity, Polarization, and Spectroscopic OCT. Transl. Vis. Sci. Technol..

[134] Enserro D.M., Demler O.V., Pencina M.J., D’Agostino R.B. (2019). Measures for evaluation of prognostic improvement under multivariate normality for nested and nonnested models. Stat. Med..

[135] Sideri O., Correa V., Ziakas N., Tsinopoulos I., Miller J.W., Vavvas D.G. (2025). Systematic Review of Proteomics in Age-Related Macular Degeneration and Pathway Analysis of Significant Protein Changes. Ophthalmol. Sci..

[136] Masli S., Sheibani N., Cursiefen C., Zieske J. (2014). Matricellular protein thrombospondin: Influence on ocular angiogenesis, wound healing and immuneregulation. Curr. Eye Res..

