# Supplementary material for: Translational Molecular and Fluid Biomarkers for Age-Related Macular Degeneration: Practical Insights from Animal Models and Humans
Source: Biomolecules. 2025 Nov 8;15(11):1571. doi: 10.3390/biom15111571 (PMC12649867; doi:10.3390/biom15111571)
Supplement: Supplementary file 1 [file biomolecules-15-01571-s001.zip › biomolecules-3886580-supplementary.pdf]

biomolecules

MDPI

Biomarker/Feature	Sample Source	Effect Size / Quantitative Measure	Diagnostic/Prognostic Performance
CFH Y402H SNP	Blood, genetic	OR 2.5 (heterozygote), OR 7.4 (homozygote)	Population attributable risk ~68%
Complement proteins (C3a, C5a, B)	Plasma	Elevated levels, fold change 1.5–3x	Associated with AMD severity
CRP	Plasma	Fold change 1.2–2x vs controls	Correlates with disease progression
IL-6, TNF-α	Plasma	Fold increases up to 2–3x in AMD	Related to disease stage
Oxidative stress markers (MDA, 8-OHdG, CEP-adducts)	Serum, plasma	CEP adducts ~60% higher in dry AMD	Correlates with disease severity
miR-126-3p	Blood	1.5–2x higher in AMD vs controls	Correlates with visual acuity
miR-155-5p	Blood	Elevated in dry AMD	Associated with disease severity
VEGF	Tear fluid	Significantly increased in wet AMD (fold change 2–4x)	Correlates with active neovascularization
Aqueous humor VEGF	Aqueous humor	Elevated levels (~2x) in neovascular AMD	Marker of angiogenesis activity
Thrombospondin 2 (THBS2)	Vitreous	Elevated in wet AMD (~2x)	Associated with fibrotic process
MMP-2, MMP-9	Retina, Bruch membrane	MMP-2 increased ~1.5–2x; MMP-9 increased 2–3x in AMD tissues	ECM remodeling and drusen formation
SMOC2	Aqueous humor	Elevated (~1.5x) in geographic atrophy	Potential marker of RPE pathology
MDA	Serum, plasma	1.5–2x higher in AMD patients	Lipid peroxidation indicator
miR-27a-3p	Blood	1.3–1.8x higher in AMD	Diagnostic potential in early and late stages
8-OHdG	Retina and aqueous humor	Significantly higher (~2x)	Oxidative DNA damage
FHR1, FHR3	Plasma	Elevated (~1.5x) in AMD severity	Modulates complement activity
IL-8	Plasma, aqueous humor	Elevated (~1.5–2x)	Indicator of CNV risk
TGF-β	Retina, plasma	Altered expression; fold change varies	Fibrosis mediator
RBP4	Serum	Increased (~1.2–1.4x) in AMD	Lipid metabolism-related biomarker
Urinary 8-OHG	Urine	Elevated (~1.8x)	Non-invasive oxidative marker

Figure S1. Summary of quantitative and qualitative biomarker metrics for AMD. A tabular overview of selected AMD biomarkers, grouped by sample source and feature type. For each biomarker, quantitative effect size estimates and diagnostic or prognostic performance metrics are provided where available. Qualitative findings and biological context are noted for markers lacking robust quantitative data. Abbreviations: SNP, single nucleotide polymorphism; AUC, area under the ROC curve; AMD, age-related macular degeneration; VEGF, vascular endothelial growth factor; N/A, not available.

Section	Model/AMD type	Source	Key biomarkers
Preclinical mouse models	Chemical ($NaIO_3$, MNU, A2E, $CoCl_2$, VEGF/FGF2)	Retina/RPE–choroid	4-HNE, 8-OHdG, nitrotyrosine, caspase-3, TNF-α, IL-6, MCP-1, MMP-2/9, TIMP-3, HIF 1α, miRNA, C3, CFH, MMP-2/9
	Chemical ($NaIO_3$, $CoCl_2$)	Plasma/Serum	C3, C4b, Factor B, SAA, IL-6, MCP-1, EPO, HIF 1α,
	$NaIO_3$ or oxidative stress models	Gut	Bacteroides Proteobacteria, Parasutterella excrementihominis
	Genetic (Ccl2$^{-/-}$, Cx3cr1$^{-/-}$, Sod1$^{-/-}$, HtrA1↑, Timp3S179C)	Retina/RPE–choroid	TNF-α, IL-1β, MCP-1, 4-HNE, 8-OHdG, MMP-2/9, VEGF, HIF-1α, ANGPT2, IRAK3, GFAP
	Genetic (C3$^{-/-}$, CFH variants)	Gut	Firmicutes, Bacteroidetes
	Laser-induced CNV	Retina/RPE–choroid	VEGFA, CCL2, CXCL9, COMP, miRNA
	Laser-induced CNV	Plasma/ Serum	miR-486a-5p, miR-92a-3p
	Laser-induced CNV	Tears / Aqueous humor	VEGF, THBS2
	Laser-induced CNV	Gut	Lachnospiraceae, Candidatus Saccharimonas
Human AMD	Dry AMD	Retina/RPE–choroid	8-OHdG, CEP, MMP-2/9, 8-OHdG, MDA, 4-HNE, DHA
	Dry AMD	Plasma/Serum	C3a, C5a, CRP, IL-6, TNF-α, miRNA
	Dry AMD	Genetics	CFH, Y402H, ARMS2/HTRA1 locus, TIMP3
	Dry AMD	Tears	Shootin-1, Histatin-3, STAT3, FGFR1
	Dry AMD	Aqueous/Vitreous	SMOC2, IL-6, clusterin, serpin A4, VEGF
	Dry AMD	Gut	Peptococcaceae, Bilophila, Roseburia, Faecalibacterium, LPS
	Wet AMD	Retina/RPE–choroid	VEGFA, CCL2, CXCL9, THBS2, MMPs
	Wet AMD	Plasma/Serum	miR-27a-3p, miR-30b, miR-191-5p, miR-145-5p, CRP, C3a/C5a
	Wet AMD	Genetics	CFH, ARMS2/HTRA1, APOE, LIPC, TIMP3 variants
	Wet AMD	Tears	VEGF
	Wet AMD	Aqueous/Vitreous	VEGF, THBS2, MMP-9
	Wet AMD	Gut	Lachnospiraceae, Bacteroidetes

Figure S2. Overview of major biomarkers identified in preclinical mouse models and human AMD, indicating model categories (chemical, genetic, laser-induced), sample sources (retina, plasma, tears, gut microbiota), and characteristic biomarkers observed in each context.
